# Participation in a High-Structure General Chemistry Course Increases Student Sense of Belonging and Persistence to Organic Chemistry

**DOI:** 10.1021/acs.jchemed.2c01253

**Published:** 2023-07-12

**Authors:** Jennifer R. Casey, K. Supriya, Shanna Shaked, Justin R. Caram, Arlene Russell, Albert J. Courey

**Affiliations:** † Department of Chemistry and Biochemistry, UCLA, Los Angeles, California 90095, United States; ‡ Center for Education, Innovation, and Learning in the Sciences, UCLA, Los Angeles, California 90095, United States

**Keywords:** Chemical Education Research, First-Year Undergraduate/General, Second-Year Undergraduate, Collaborative/Cooperative Learning, Student-Centered Learning, Minorities in Chemistry, Women in Chemistry

## Abstract

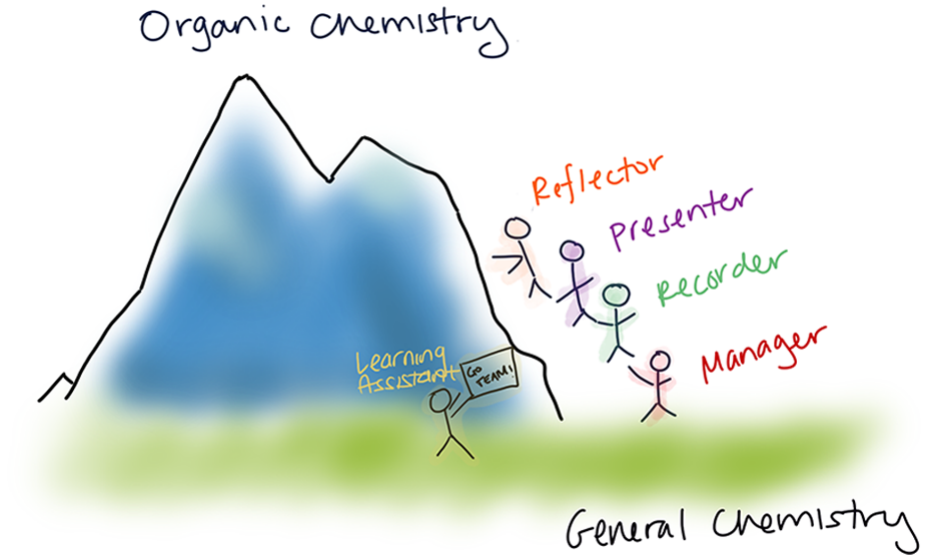

A parallel series of general chemistry courses for Life Science Majors was created in an effort to support students and improve general chemistry outcomes. We created a two-quarter enhanced general chemistry course series that is not remedial, but instead implements several evidence-based teaching practices including Process Oriented Guided Inquiry Learning (POGIL), Peer-Led Team Learning (PLTL), and the Learning Assistant (LA) model. We found that students who took enhanced general chemistry had higher persistence to the subsequent first organic chemistry course, and performed equally well in the organic course compared to their peers who took standard general chemistry. Students in the first enhanced general chemistry course also reported significantly higher belonging, although we were unable to determine if increased belonging was associated with the increased persistence to organic chemistry. Rather we found that the positive association between taking the enhanced general chemistry course and persistence to organic chemistry was mediated by higher grades received in the enhanced general chemistry course. Our findings highlight the responsibility we have as educators to carefully consider the pedagogical practices we use, in addition to how we assign student grades.

## Introduction

General chemistry, a lower-division requirement for many STEM majors, can pose difficulties for the nonchemistry STEM major.^1^ The high proportion of Ds, Fs, Withdrawals, and Incompletes (DFWIs) in these courses leads general chemistry to fall under the moniker “weeder” course.^2^ Given that earning even one DFWI in an introductory STEM course is a strong predictor of switching to a non-STEM major, it is particularly concerning that NALA (Native Hawaiian/Pacific Islander, American Indian/Alaska Native, Latinx/Hispanic, and African American/Black) students receive a disproportionately larger number of these grades compared to their WA (White and Asian/Asian American) peers.^3^ This fact may partially explain why, despite similar levels of declared interest in becoming STEM majors, NALA students are found to have higher STEM attrition rates.^4−7^ While there are many reasons behind a student’s choice to leave a STEM major (such as discovering an interest and/or aptitude for a non-STEM field), we must acknowledge the fact that some students feel pushed out of STEM and ultimately find refuge elsewhere.^8−11^

A student’s lack of success in general chemistry has often been partially attributed to inadequate high school preparation. Even at highly selective colleges and universities, students enter these courses with a wide variation in prior chemistry experience: some students have taken Advanced Placement (AP) chemistry, while others have had no exposure to chemistry at all. The opportunity to take a high-quality college-level chemistry course while in high school is associated with race and socioeconomic class due to racial segregation and inequities in school funding.^11−13^ In other words, due to systemic racism, students who enter general chemistry courses with less prior chemistry experience tend to identify as NALA and/or are from low socioeconomic status backgrounds. But this alone does not explain the disparities we see in grades and attrition between NALA and WA students; it has been shown that significant attrition gaps persist even when prior preparation is accounted for.^3,4,14^ And even those students who would be characterized as “prepared” (i.e., they have taken advanced coursework in high school) can report feeling ill-equipped to handle the challenges of college STEM coursework.^2,15^

## Rationale and Background

Often programs designed to support students derive from a deficit model of academic opportunities.^5,16^ One such intervention is participation of students in academic support programs that are separate from, but coordinated with, their course work. These programs employ a holistic approach that may include counseling, collaborative learning workshops, and/or exposure to research.^17,18^ These programs, however, often require an application process and early commitment (e.g., the summer before starting college), along with an investment of time that may discourage participation by students with multiple demands on their time including family obligations and the need to support themselves financially. Additionally, other criteria for such academic support programs may be related to high school GPA or SAT score and thus exclude such support for all needful students.

Rather than trying to approach this situation from a student-deficit perspective through means such as academic support programs or remedial coursework, one can approach this problem from a course-deficit perspective and consider what changes can be made to improve the experiences of students in our introductory STEM courses.^2,5,17^ For instance, incorporating active learning in the classroom is associated with both increased learning^19^ and decreased disparities in exam scores and passing rates for low-income students as well as students who identify as NALA.^20^ Highly structured courses have been associated with similar effects.^21,22^ But these and other study findings suggest that active learning needs to be done in relatively intense and deliberate ways in order to see a decrease in performance differences.^20,23^

Three nationally adopted instructional innovations have been shown to integrate this additional structure into the chemistry classroom:

### Process Oriented Guided Inquiry Learning (POGIL, https://pogil.org)^24−26^

In a POGIL classroom, students work in small teams on highly structured worksheets organized around an explore–invent–apply learning cycle. Teams usually consist of 3–5 students and each team member is assigned a specific role. Through this collaborative work, students construct their own knowledge while simultaneously developing process skills (e.g., teamwork, management, information processing) that have the potential to benefit all learning.^27^ POGIL activities frequently take the place of traditional lectures, and the instructor acts as a facilitator of critical thinking rather than as the presenter of knowledge. For this reason, POGIL is often used in smaller classroom settings of 30 students or fewer, although it has been successfully implemented in large lecture courses as well.^30^ Integrating POGIL can lead to increased performance on standardized exams from the American Chemical Society,^28^ along with growth in process skills.^29^

### Peer-Led Team Learning (PLTL, https://sites.google.com/view/pltl)^31−33^

Rather than replace lecture time with group work, undergraduate students who were previously successful in a course can act as peer leaders of workshops that are supplemental to yet well-integrated with the course. The groups tend to be larger (6–8 students), and the peer leader creates a supportive environment to encourage all students to actively participate in the problem-solving sessions. PLTL is based upon the Zone of Proximal Development theory,^34^ which emphasizes the benefits of learning from a capable peer. Consequently, an instructor is generally not present during the workshops and as such, peer leaders undergo intensive training in leadership and group facilitation. There are many recorded benefits of employing PLTL in STEM such as improved course performance, increased retention, and positive student perceptions.^35−37^

### Undergraduate Learning Assistant (LA) Program (https://www.learningassistantalliance.org)^38,39^

The LA program also uses undergraduates who are trained to foster collaborative and inclusive learning during class. While having a similar structure to PLTL, a major difference is that rather than separate workshops, LAs are integrated into the classroom and provide assistance to the instructor. Use of LAs is also associated with increased student satisfaction,^40^ decreased failure rates,^41^ increased performance on higher-order assessments,^18^ and more equitable classrooms.^42^

## Details of Intervention

Given that our institution has documented grade disparities between NALA and WA students in our general chemistry track for Life Science majors, we developed a parallel series of enhanced general chemistry courses that uses the three above-mentioned high-impact practices shown to support students. It should be noted that the enhanced courses are not remedial, and the learning objectives are identical to those used in the standard general chemistry courses offered to all Life-Science-focused students.

The standard and enhanced versions of general chemistry both consist of three 50 min lectures a week. The courses are similar in size (approximately 300 students in a standard lecture relative to 230 students in an enhanced lecture), and both lectures are assigned four graduate Teaching Assistants (TAs). The primary difference occurs in discussion section. While the standard series has a 50 min weekly discussion section, the weekly discussion time has been increased to 110 min for the enhanced series. Both the standard and enhanced discussion sections enroll between 20 to 25 students. With the extra time designated in the enhanced discussions, we are able to structure the sections around evidenced-based practices, specifically PLTL and POGIL. Essentially the discussion sections are centered on POGIL-based worksheets, and facilitated by LAs who integrate techniques from PLTL. We are not the first to blend these methodologies,^35,43^ but our particular approach is outlined in Table 1 and in the SI. By increasing the discussion time, we are able to build intentional teamwork into the classroom culture. While there is more class time associated with the enhanced series, both the standard and enhanced general chemistry series carry the same 4 units of course credit. The expectation is that time students would normally use on independent study can instead be used on the guided development of chemistry concepts and process skills.

**Table 1:** Pedagogical Elements of the Enhanced General Chemistry Course

Course Information	Description	Pedagogy Used
General Setup	Lectures are retained	PLTL/LA
	Group work is incorporated into mandatory discussion sections	POGIL/LA
	TA is present during discussion section	POGIL/LA
Group Structure	Each team is assigned an undergraduate Learning Assistant who promotes group interactions	PLTL/LA
	Students are assigned to a permanent team	POGIL
	Teams consist of 3–4 students	POGIL
	Each team member is assigned a role that rotates weekly	POGIL
Group Activities	Teams meet each week in discussion, where they work on and complete a structured activity focused on the learning cycle	POGIL
	Midterm exams are two-stage,^104^ with a second group attempt	PBL^a^
Responsibilities	Students complete a preactivity prior to discussion	POGIL
	LAs spend 3 h each week in preparatory meetings	PLTL/LA
	TAs support teams and facilitate larger group discussion	POGIL
	Instructor creates weekly activities and anticipates facilitation needs	POGIL/LA
	Instructor prepares TAs and LAs for facilitation of activity	PLTL/LA

a This type of testing is more reminiscent of Problem Based Learning (PBL),^43^ as POGIL PLTL, and the LA model do not promote specific testing strategies.

## Theoretical Frameworks

The design of the enhanced series was grounded in self-determination theory (SDT). SDT assumes that people are inherently interested in gaining knowledge due to an intrinsic curiosity about the world. As educators, we can lobby this intrinsic motivation for learning by promoting student autonomy, focusing on learning goals rather than performance goals, building student self-efficacy through mastery experiences with feedback, and encouraging relevance and relatedness in the classroom.^44−46^ It has been found that having supportive instructors and peers is positively correlated with students’ perceived competence and intrinsic motivation, which in turn can lead to greater academic achievement and lower rates of attrition.^47^ For this reason, the enhanced series incorporates multiple, high-impact practices that have been shown to support students in these ways (see Details of Intervention for more information).

SDT is focused on exploring the connection between a student’s tendency toward growth and the potential causes for resiliency. Our research questions are centered on this connection, specifically how persistence in STEM is related to sense of belonging and grade received. The connection between these three variables was introduced in Tinto’s model of retention, which posits that college attrition is related to a student’s personal attributes and experiences, as well as their social and academic integration within the college community.^48^ Social integration is related to involvement in activities as well as positive relationships with peers and faculty (i.e., sense of belonging); academic integration is related to student academic performance, of which grades are one measure. Studies have shown a positive relationship between sense of belonging and persistence,^49,50^ including a recent report that explores sense of belonging and continuation in general chemistry.^51^ A large body of literature also demonstrates the impact of grades on persistence, thus driving our investigation into this effect.^2,3,52,53^

## Research Questions

Both the standard and the enhanced general chemistry sequences track all students into the same standard organic chemistry courses, allowing us to compare various student outcomes across standard and enhanced general chemistry offerings. Our investigation was centered on three primary research questions:

1. Compared to taking a standard general chemistry course, to what extent is taking an enhanced general chemistry course associated with
  a. increased student persistence to the first organic chemistry course in the series?
  b. increased student grades in the first organic chemistry course in the series?
2. To what extent does taking an enhanced general chemistry course mitigate disparities in persistence based on race/ethnicity and sex?
3. To what extent does taking an enhanced general chemistry course improve student outcomes in the general chemistry series
  a. through increased student sense of belonging?
  b. through students receiving higher grades in the enhanced general chemistry course?

## Methods

### Positionality of Authors

We acknowledge that the identities and experiences of researchers influence their work, both implicitly and explicitly. We come into this work in various ways. Many of us (AC, AR, JCaram, and JCasey) are chemistry and biochemistry faculty who hold PhDs in these fields. AC and JCaram are research faculty; AR and JCasey are instructional faculty. Two of us (KS and SS) are educational developers with STEM PhDs. AR, KS, SS, and JCasey have prior experience with discipline-based education research. Our social identities include women (AR, KS, SS, JCasey), men (AC, JCaram), Latinx (JCaram), South Asian (KS), Middle Eastern (AC), and White (AR, SS, JCaram, JCasey).

### University Information

This study occurred at a large research-intensive public university in the western United States. The university is on the quarter system, with each academic year consisting of three-quarters: Fall (F), Winter (W), and Spring (S). In addition, there is an optional Summer (Su) quarter. The demographic breakdown of the incoming student population in 2020 was 3% African American, <1% American Indian and Alaskan Native, 33% Asian, <1% Pacific Islander, 21% Hispanic, and 25% White; 33% of students are first-generation college students and 50% of students receive need-based financial aid.

### Chemistry Series for Life-Science Majors

Since 1998, the university has offered a biologically focused, four-quarter general and organic chemistry series for Life Science (LS) majors. Because all LS majors require both the general chemistry and organic chemistry courses in this series for degree completion, we expect students who enroll in the LS general chemistry series to eventually enroll in the organic series. The timing in which students begin as well as move through the series varies greatly as each course is offered every quarter, including during summer. LS students are not required to start the series during their first quarter, but approximately 40% do.

Each Fall quarter, around 1200 students enroll in the first course of the general chemistry series for Life Science majors (GChem1). Generally, the multiple sections consist of approximately 300 students each and are taught by two instructors. While the instructors set up their own courses (e.g., syllabus, resources, quizzes, exams, etc.), a general set of agreed upon learning objectives are used by all instructors teaching the course. More information on the setup of lecture and discussion can be found in the SI. This study includes the classes taught by three instructors (I1, I2, and I3) who have been responsible for teaching both the standard and enhanced sections of GChem1 since Fall 2017.

General chemistry is a two-quarter series, and both quarters are prerequisites for organic chemistry. While students who complete GChem1 in the Fall are not required to enroll in the second course of the general chemistry series (GChem2) the following Winter quarter, approximately 80% of students do. Additionally, the vast majority of students did not switch between the standard and enhanced general chemistry series (94% for the enhanced series, 96% for the standard series) when going from GChem1 to GChem2. While our investigations focused on GChem1, many of the students who took enhanced GChem1 also took enhanced GChem2.

### Organic Chemistry

The first organic chemistry course in the series (OChem1) is taught by many different instructors with agreed upon topics but varying course setups.

### Participants

Our cohort comparison study uses data collected during the Fall 2017 (F17), Fall 2018 (F18), Fall 2019 (F19), and Fall 2020 (F20) quarters (see Table S1 for enrollment details). Given that the study was conducted after the courses had ended and that collected data came from curriculum-related activities, the university’s Institutional Review Board approved the use of all participants’ data given adequate deidentification; participant consent was not required (IRB#21–001162). The F17–F19 data corresponds to standard lecture sections of GChem1 taught by I1, I2, and I3 while the F20 data consists of one enhanced lecture section of GChem1 taught by I1 and three standard lecture sections of GChem1 taught by I2. All sections in F17, F18, and F19 were taught in-person, while all sections (both standard and enhanced) in F20 were taught entirely remote due to the COVID-19 pandemic.

### Selection Criteria for Fall 2020 Enrollment

In Fall 2020, students were given a recommendation as to which version of GChem1 to enroll in based on an optional chemistry diagnostic exam which included questions on prior chemistry experience as well as mathematical reasoning and logical thinking.^54^ This was merely a recommendation and we found that only 55% of the students who were recommended to take the enhanced series did so. This is in contrast to the 78% of students who acted in accordance with their recommendation to enroll in the standard GChem1 course. The average score on the diagnostic exam for the enhanced series was 3.00 versus 3.27 for the standard general chemistry series (note that only 58% and 81% of students enrolled in standard and enhanced general chemistry completed the diagnostic exam respectively). This means that students enrolled in the enhanced series were predicted to earn DFWIs at higher rates relative to their peers in the standard series.

### Demographics

Student demographics (sex, race/ethnicity, SAT score, and high school GPA) were obtained with IRB approval from the university’s registrar’s office. The registrar’s data included three sex options: male, female, and neither male nor female designated as X. Ideally, we would have used data on gender identity as well since the social construct of gender shapes people’s experiences, however, institutional data did not include gender. Students can self-identify ethnicity as Hispanic/Latino, and can choose from multiple race options: African American/Black, American Indian/Alaska Native, Asian, Native Hawaiian/Pacific Islander, and White. Because of our limited sample sizes (especially for the enhanced chemistry section in Fall 2020), we combined all students who self-identified as Native Hawaiian/Pacific Islander, American Indian/Alaska Native, Latinx/Hispanic, and African American/Black under the category of NALA. These groups are all known to be underrepresented in STEM compared to their overall populations in the US.^55^

### Preparation

We used SAT math score and high school GPA as predictors of preparation. While these are both imperfect measures of students’ prior experience with chemistry, they are correlated and serve as a useful proxy.^56,57^ We had SAT math scores for 77% of our student population and high school GPA for 98% of our student population.

### Social Belonging

A six-item validated survey was used to measure social belonging (see SI).^51,58^ The survey consists of two measures: *perceived belonging* (four items) and *belonging uncertainty* (two items). The statements were assessed on a six-point Likert scale: strongly disagree, disagree, mildly disagree, mildly agree, agree, strongly agree. *Perceived belonging* relates to a student’s general feelings of belonging in the course in relation to their peers and instructor while *belonging uncertainty* focuses on the stability of a student’s sense of belonging, as well as the effect performance can have on it. The belonging survey was administered in Fall 2020 to both standard and enhanced GChem1 sections during the first and last weeks of the quarter. While both instructors asked students to complete the survey (administered online via Google Surveys), only the enhanced section of general chemistry was offered points for completing this survey or an alternative. As a result, the response rate to the belonging survey was 92.7% in the enhanced section but only 29.2% in the standard section.

### Persistence

**Figure 1:**
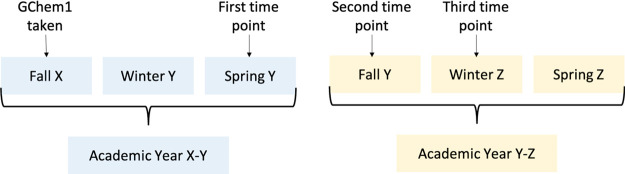
Outline of how persistence was measured in this study. Only students who enrolled in GChem1 during Fall quarter were included. Persistence was measured over three time points. **First time point:** Students who took OChem1 during the Spring quarter (Spring Y) or Summer session of the same academic year in which they took GChem1. **Second time point:** Students who took OChem1 during the following Fall quarter (Fall Y) of the subsequent academic year. **Third time point:** Students who took OChem1 during the following Winter quarter (Winter Z) of the subsequent academic year. These time points are cumulative.

Persistence to the first organic chemistry course in the series was coded as a binary variable. Delaying completion of OChem1 is not necessarily an indication that a student is struggling with the chemistry series. Thus, we looked at the enrollment in OChem1 over three time points (see Figure 1): the Spring quarter in the same academic year in which they took general chemistry (Spring Y), the following Fall quarter during the subsequent academic year (Fall Y), or the following Winter quarter during the subsequent academic year (Winter Z). These time points are cumulative, meaning that the number of students who enroll in OChem1 by the third time point includes students who enrolled in OChem1 during the two previous time points as well. The Life Science division recommends students take OChem1 no later than four quarters (excluding summer) after taking GChem1. As such, students who waited to enroll in OChem1 in the following Spring quarter of the subsequent academic year (Spring Z) or later were excluded from the study. The small number (6%) of students who enrolled in OChem1 during the summer session were included in the study, and were grouped with the previous Spring cohort.

### Data Analysis

All analyses were run using open-source software *R*(59) in RStudio using packages ggplot2,^60^ tidyr,^61^ sjPlot,^62^ gtsummary,^63^ patchwork,^64^ lavaan,^65^ and mediation.^66^ Depending on the research question being investigated, we employed logistic regression, multiple linear regression, confirmatory factor analysis, and mediation modeling techniques.

We used logistic regressions to assess the association between GChem1 course type taken in Fall X (i.e., standard or enhanced) and student persistence to OChem1 at three time points: i. Spring/Summer Y, ii. Fall Y, iii. Winter Z (see Figure 1). In addition to course type, we included the following covariates in our model: instructor, term when the course was taken (F17, F18, F19, or F20), *z*-score of SAT math score, and *z*-score of high school GPA.

To assess the association between general chemistry course type and student grades in OChem1, we first converted student letter grades to numeric values (A+/A = 4.0, A– = 3.7, B+ = 3.3, B = 3.0, B– = 2.7, C+ = 2.3, C = 2.0, C– = 1.7, D+ = 1.3, D = 1.0, D– = 0.7, NP/F = 0) and then used multiple linear regression models. Given that grading schemes can vary significantly across instructors, our comparison cohorts were for students who took general chemistry in Fall 2020 (when both the standard and enhanced series were offered) since these students went on to take OChem1 courses with the same instructors for each of the three time points. We used the grades of students that persisted to OChem1 by the third time point as the outcome and general chemistry course type as the predictor for this analysis. We included the *z*-score of SAT math score, high school GPA, and the term in which OChem1 was taken as covariates in this model. In order to assess whether taking the enhanced course has differential associations with outcomes for students with different identities, we added demographic variables (sex and race/ethnicity) along with interactions between course type and sex and course type and race/ethnicity as predictors into our logistic regression and linear regression models described above.

We validated the two-factor structure of the sense of belonging scale for our population using a confirmatory factor analysis.^58,67^ The CFA indicated an acceptable fit based on Comparative Fit Index (CFI = 0.99 for predata and 0.98 for postdata, >0.95 indicates good fit), Root Mean Square Error of Approximation (RMSEA = 0.04 for predata and 0.07 for postdata, ≤0.06 indicates good fit), and Standardized Root Mean Square Residual (SRMR = 0.02 for predata and postdata, ≤0.08 indicates good fit). We then calculated factor scores for pre- and post- *perceived belonging* and *belonging uncertainty* and used those in further analyses. To compare the pre- and post- difference in *perceived belonging* and *belonging uncertainty* between the two course types, we used multiple linear regressions with post-sense-of-belonging factor scores as the outcomes and pre-sense-of-belonging factor scores and course type as the predictors. We also included the *z*-score of high school GPA and SAT math score as covariates in these models. In addition, we examined associations between change in sense of belonging and students’ social identities (sex and race/ethnicity).

Since SAT data was missing for many students, we repeated all regression models without SAT math score as a predictor on a larger data set that still included high school GPA. Those results can be found in the SI.

Finally, we used mediation modeling^68^ to assess whether students’ grades in GChem1 mediate the association between the type of GChem1 course taken and persistence to OChem1. To estimate the mediation effect, we ran two models: (i) the “mediator model” with numeric grade in GChem1 as the outcome and type of GChem1 course as the predictor, and (ii) the “outcome model” with persistence to OChem1 as the outcome and the type of GChem1 course and numeric grade in GChem1 as the predictors. We included instructor, term, SAT math score, and high school GPA as covariates in both models. With these two models as inputs, the “mediate” function calculated the estimated mediation effect using 1000 quasi-Bayesian Monte Carlo simulations to calculate confidence intervals and statistical significance. We repeated these analyses for all three time points. We were unable to assess whether students’ sense of belonging at the end of GChem1 mediates their persistence to OChem1 because of nonequivalence of the subsets of students who completed the survey in our comparison cohorts. The subset of students who took standard GChem1 in Fall 2020 and filled out the sense of belonging survey was biased toward students who received higher grades (3.38 compared to 3.23 for all standard GChem1 Fall 2020 students) and persisted to OChem1 at higher rates (87% compared to 82% by the third time point).

## Results and Discussion

### Research Question 1a: Taking the Enhanced General Chemistry Course Increases Student Persistence to the First Organic Chemistry Course in the Series

Our results show that taking the first enhanced general chemistry course was associated with a greater likelihood of taking the first organic chemistry course in the series at various time points (Table 2). This effect is most pronounced when we look at persistence by the second time point; the persistence of students in enhanced GChem1 to OChem1 by the following Fall quarter is similar to the persistence of students in standard GChem1 by the following Winter quarter (a quarter later). This result is particularly remarkable given that the percentage of students in the enhanced course had less prior academic preparation based on SAT math score, chemistry diagnostic score, and prior chemistry preparation (Table S1).

**Table 2:** Student Persistence to the First Organic Chemistry Course in the Series at Various Time Points after Taking the Standard (S) or Enhanced (E) General Chemistry Course^a^

	S F17 I1	S F17 I2	S F18 I2	S F18 I3	S F19 I2	S F19 I3	S F20 I2	E F20 I1
*N*	341	588	883	244	893	156	919	233
Took OChem1 by first time point	163 (47.8%)	316 (53.7%)	367 (41.6%)	120 (49.2%)	494 (55.3%)	76 (48.7%)	494 (53.5%)	136 (58.4%)
Took OChem1 by second time point	238 (69.8%)	441 (75.0%)	603 (68.3%)	186 (76.2%)	691 (77.4%)	109 (69.9%)	687 (74.5%)	191 (82.0%)
Took OChem1 by third time point	266 (78.0%)	488 (83.0%)	703 (79.6%)	202 (82.8%)	749 (83.9%)	128 (82.1%)	755 (81.9%)	199 (85.4%)
Passed OChem1 with C or higher by third time point	259 (76.0%)	458 (77.9%)	684 (77.5%)	187 (76.6%)	742 (83.1%)	123 (78.8%)	707 (76.9%)	185 (79.4%)

a I1, I2, and I3 indicate Instructors 1, 2, and 3 respectively.

**Figure 2:**
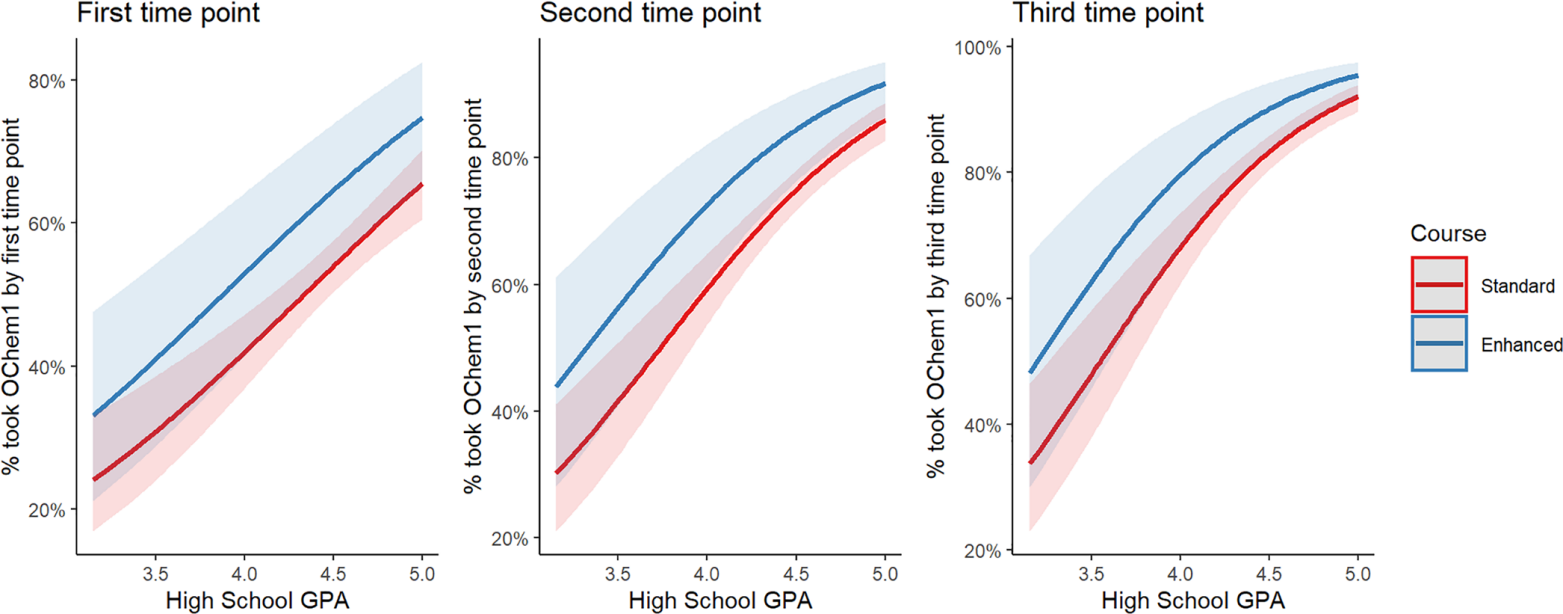
Predicted values of % student persistence to the first organic chemistry course in the series at three different time points based on logistic regression models with GChem1 course as the predictor. The models controlled for term, instructor, SAT math score, and high-school GPA.

The positive association between taking the enhanced general chemistry course and persistence to the first organic chemistry course in the series remains even after controlling for instructor, Fall quarter when GChem1 was taken, and *z*-scores of SAT math score and high school GPA (Figure 2, Table S2). While the effect size was large for all three time points (log(Odds Ratio) = 0.44, 0.59, and 0.60, respectively), the effect was found to be statistically significant at the second time point (*p*-value = 0.06, 0.03, and 0.06, respectively). We repeated these analyses with the larger data set that included high school GPA data but not SAT math score data and found similar effect sizes (log(Odds Ratio) = 0.43, 0.73, and 0.60, respectively). However, in this data set the effect of taking enhanced GChem1 is statistically significant at all three time points (*p*-value = 0.035, 0.003 0.031 respectively), likely due to greater statistical power associated with the larger sample size (Figure S1, Table S3).

While it is promising that taking the enhanced GChem1 course is associated with increasing Life Science majors’ persistence to organic chemistry, the fact that the data indicates more students are taking the first organic chemistry course by the following Fall quarter is also an important finding. Many LS majors who intend to take the MCAT hope to do so in their junior year, making it important to complete their general chemistry, organic chemistry, and biochemistry courses by the end of their sophomore year.^69^ Taking the first organic chemistry course in the series during the following Fall quarter makes this possible. Furthermore, there have been discussions about the importance of exposing Life Science majors to organic chemistry concepts earlier in their academic career given the importance of organic chemistry concepts to the biological sciences.^70,71^

### Research Question 1b: Students Who Took the Enhanced General Chemistry Course Earned a Similar Grade in Their First Organic Chemistry Course Compared to Students Who Took the Standard General Chemistry Course

**Figure 3:**
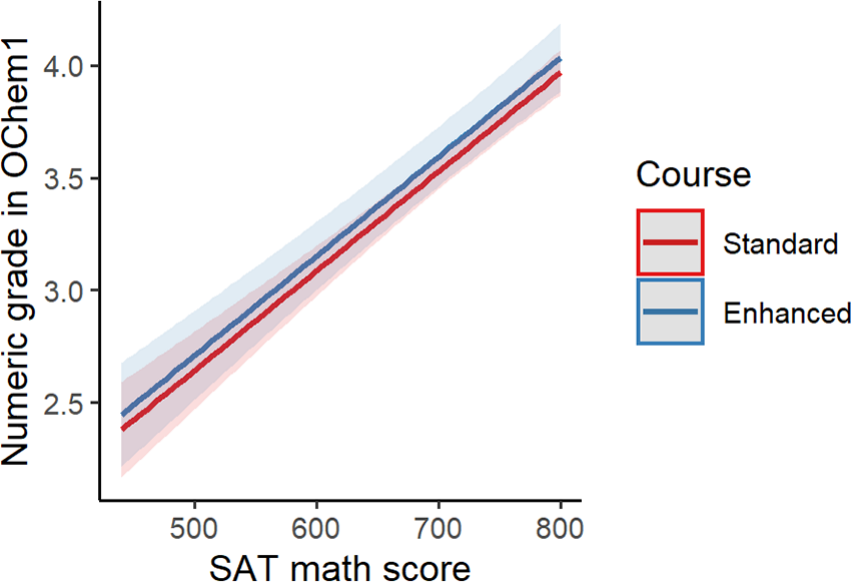
Predicted grades in the first organic chemistry course in the series based on linear regression models with GChem1 course as the predictor. The models controlled for the term in which organic chemistry was taken, SAT math score, and high-school GPA.

There was no association between the grades received by students in the first organic chemistry course of the series and whether or not they took the first enhanced general chemistry course when controlling for SAT math score and high school GPA (Figure 3, β = 0.07 (95% CI: −0.07,0.21), *p*-value = 0.4, Table S4). We repeated these analyses with the larger data set that included high-school GPA data but excluded SAT math score data and found similar results (Figure S2, β = −0.01 (95% CI: −0.14,0.12), *p*-value = 0.9, Table S5). Together with the previous results, this means that students taking the enhanced general chemistry course persist to organic chemistry at a higher rate and perform similarly to their peers.

When considering race/ethnicity and/or sex, there were no significant differences in student grades in the first organic chemistry course based on whether a student took the standard or enhanced general chemistry course. Among students who took the standard GChem1 course, NALA students had a mean OChem1 grade of 3.12 ± 0.91 SD compared to 3.67 ± 0.63 SD for WA students. Similarly, among students who took the enhanced GChem1 course, NALA students had a mean OChem1 grade of 3.07 ± 0.97 SD compared to 3.64 ± 0.66 SD for WA students. This is in contrast to the grades received in GChem1, where NALA and WA students in the enhanced GChem1 course saw a smaller GPA difference (0.51 compared to 0.91 in the standard GChem1 course). The effects of the higher grades received in enhanced GChem1 are further explored under Research Question 3b.

The fact that taking the enhanced GChem1 course did not lead to improved course grades in OChem1 is worth considering. One potential explanation is that the content emphasized in GChem1 and OChem1 is different, even though there are some commonalities such as molecular shape, hybridization, and resonance. But it was our hope that the process skills emphasized in the enhanced series (e.g., information processing, critical thinking) would transfer over to future courses. It should be noted that while the organic chemistry courses sometimes use Learning Assistants in discussion sections, these courses are generally much less structured and do not implement other high-impact practices, such as PLTL or POGIL. Ideally the benefits of the enhanced GChem1 course would carry over to OChem1 given the resources and efforts required to completely transform the GChem series, but that does not seem to be the case. This then suggests that isolated interventions may not be enough for long-term support of students and that ultimately we need to continue these practices beyond first year STEM courses. Evidence does exist for the transferability of general and contextualized skills, but this may require continued sociocultural support.^72^

### Research Question 2: Students with Different Social Identities Benefitted Similarly from Taking the Enhanced General Chemistry Course

**Figure 4:**
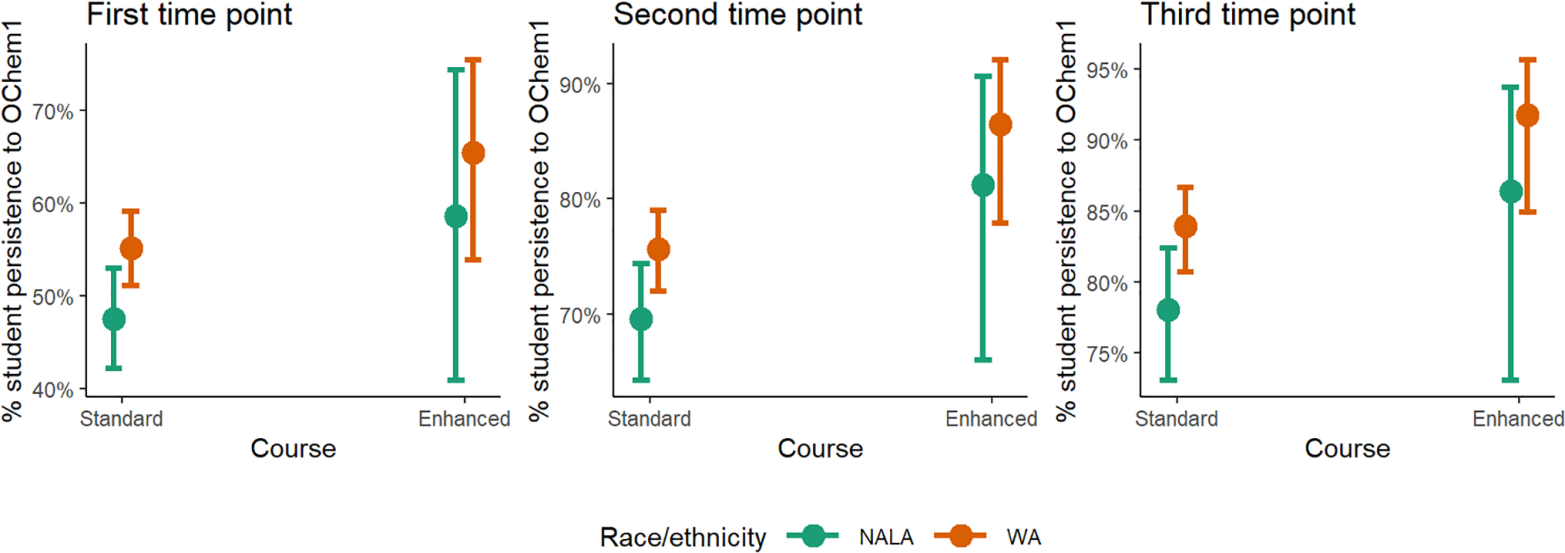
Predicted values of % student persistence to the first organic chemistry course in the series at three different time points based on logistic regression models with GChem1 course and race/ethnicity (classified as NALA: Native Hawaiian/Pacific Islander, American Indian/Alaska Native, Latinx/Hispanic, and African American/Black; or WA: White or Asian/Asian American) as predictors. The models controlled for term, instructor, sex of the student, SAT math score, and high-school GPA.

**Figure 5:**
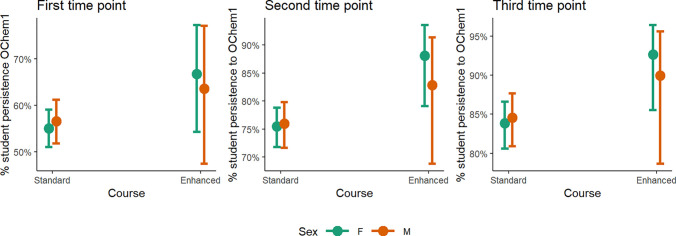
Predicted values of % student persistence to the first organic chemistry course in the series at three different time points based on logistic regression models with GChem1 course and sex (classified as F: female or M: male) as the predictor. The models controlled for term, instructor, race/ethnicity, SAT math score, and high-school GPA.

Taking the enhanced general chemistry course improved persistence to the first organic chemistry course in the series similarly for both NALA and WA students, as well as for both male and female students (Figures 4, 5, S3, and S4, Tables S6 and S7). For NALA students enrolled in the enhanced GChem1 course, 50% enrolled in OChem1 by the first time point, 75% by the second time point, and 77% by the third time point. This is compared to 43%, 63%, and 71% for the standard GChem1 course. Persistence to OChem1 is similar at all three time points for male and female students, and is consistently higher for students who took enhanced GChem1 compared to students who took standard GChem1. The increased retention for NALA and female students mirrors the positive outcomes previously seen from implementing PLTL^73−75^ as well as using LAs.^42^

Taking the enhanced general chemistry series did not however eliminate the disparities between NALA and WA students who persisted at higher rates; there was no statistically significant interaction between NALA status and the type of general chemistry course taken (log(Odds Ratio) = 0.00 (95% CI: −0.75,0.75), *p*-value >0.9 for the model with both SAT math score and high school GPA and log(Odds Ratio) = −0.12 (95% CI: −0.81,0.57), *p*-value = 0.7 for the model with only high school GPA, see full regression model results in Tables S8 and S9). Even among students that took the enhanced course, the disparities seen in persistence to OChem1 between NALA and WA students was about 11% at the first time point, 8% at the second time point, and 10% at the third time point compared to 9%, 13%, and 12%, respectively, among students that took standard GChem1. It appears that taking enhanced GChem1 increased persistence uniformly, without any particular benefit to NALA students compared to WA students. This finding is in line with what has been found by other studies, namely that high-impact practices may not necessarily eliminate equity gaps for students traditionally underserved by higher education STEM structures.^42,76−78^

### Research Question 3a: Students Who Took the Enhanced Course Showed an Increase in Perceived Belonging and a Decrease in Belonging Uncertainty at the End of the Fall 2020 Quarter

**Figure 6:**
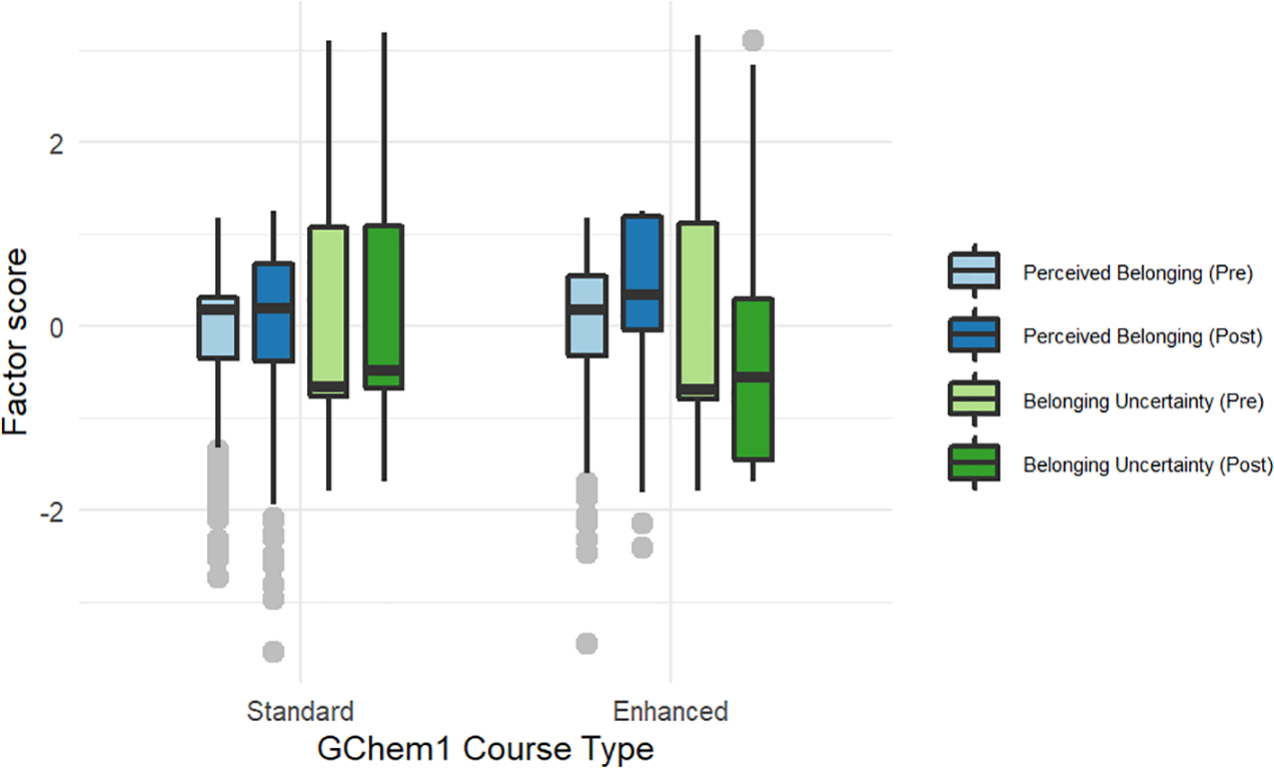
Boxplots of pre- and post- *perceived belonging and belonging uncertainty* factor scores for the standard and enhanced GChem1 courses. A higher factor score for *perceived belonging* is associated with a higher sense of belonging, while a lower factor score for *belonging uncertainty* is associated with a higher sense of belonging.

Students who took enhanced GChem1 showed an increase in *perceived belonging* (mean factor score of 0.37 at the end of the quarter compared to 0.06 at the beginning). By contrast, students who took standard GChem1 showed no substantial change in their *perceived belonging* (mean prefactor score of 0.03 and postfactor score of 0.05). Enhanced GChem1 students also showed a decrease in *belonging uncertainty* over the course of the quarter (mean prescore of −0.08 compared to mean postscore of −0.37), while Standard GChem1 students showed no meaningful difference in belonging uncertainty (mean prescore of −0.03 compared to mean postscore of 0.03). These effects were statistically significant when controlling for pre-belonging score as well as high school GPA and SAT math score (Figure 6, Tables S10 and S11; for models with both SAT score and high school GPA: perceived belonging enhanced GChem1 β = 0.34 (95% CI: 0.17,0.50), *p*-value <0.001; belonging uncertainty enhanced GChem1 β = −0.32 (95% CI: −0.51,-0.14), *p*-value <0.001). These results are conservative given that the response rate to the belonging survey was 92.7% for enhanced GChem1 and 29.2% for standard GChem1, with students who completed the survey in GChem1 having received higher grades and persisted to OChem1 at higher rates. The biased sample from standard GChem1 suggests that the observed differences in belonging between standard and enhanced GChem1 are likely underestimated.

We disaggregated the data by race/ethnicity and sex and found that generally female NALA students had the lowest reported *perceived belonging* (−0.13 ± 0.79 in the standard course and −0.08 ± 0.91 in the enhanced course) and the highest reported *belonging uncertainty* (0.19 ± 1.28 in the standard course and 0.36 ± 1.37 in the enhanced course) prior to starting general chemistry, indicating that female NALA students began the chemistry series feeling less confident about their belonging relative to their peers (Table S12). Conversely, male WA students had presurvey scores that indicated they entered GChem1 with the strongest feelings of belonging in chemistry (perceived belonging: 0.13 ± 0.59 in the standard course and 0.17 ± 0.91 in the enhanced course; belonging uncertainty: −0.23 ± 1.01 in the standard course and −0.41 ± 1.36 in the enhanced course). These data align with previous findings.^79−82^ There was no statistically significant interaction between the type of GChem1 course taken and sex or race/ethnicity on students’ *perceived belonging* at the end of the course (for models with both SAT math score and high school GPA: enhanced GChem1*Male β = 0.31 (95% CI: −0.06,0.68), *p*-value = 0.10 and enhanced GChem1*NALA β = 0.17 (95% CI: −0.24,0.59), *p*-value = 0.4, Tables S13 and S14). Similarly, there was no statistically significant interaction between the type of GChem1 course taken and sex or race/ethnicity on students’ *belonging uncertainty* at the end of the course (for models with both SAT math score and high school GPA: enhanced GChem1*Male β = −0.21 (95% CI: −0.62,0.21), *p*-value = 0.3 and enhanced GChem1*NALA β = 0.05 (95% CI: −0.42,0.51), *p*-value = 0.8, Tables S13 and S14). In other words, taking enhanced GChem1 did not disproportionately benefit NALA students or female students after controlling for pre-belonging score, high-school GPA, and SAT math score.

Looking more closely at the raw data for the different belonging measures, we noticed that in the standard GChem1 course, WA students (both male and female) showed a very slight increase in *perceived belonging* by the end of the course, while NALA students (both male and female) showed a noticeable decrease in *perceived belonging*. By contrast, all students in the enhanced GChem1 course showed an increase in *perceived belonging*. This increase is likely to be an underestimate because standard GChem1 students who completed the sense of belonging survey were skewed toward students doing well in the course. In terms of *belonging uncertainty*, all standard GChem1 students except WA females reported an increase in *belonging uncertainty*, with the largest increase being observed for NALA males. Students enrolled in the enhanced GChem1 course all reported a decrease in *belonging uncertainty*, with both WA and NALA females having relatively larger decreases and NALA men having a more modest decrease.

The positive impact the enhanced course had on all students’, including NALA students’, sense of belonging is noteworthy given how high sense of belonging has been linked to many beneficial outcomes, including increased motivation and self-efficacy,^83^ greater STEM persistence,^50,51,79^ and better academic performance and health outcomes.^84^ Although an increase in sense of belonging is often associated with specific social-psychological interventions (such as implementing value affirmation exercises),^85^ no such interventions were implemented in the enhanced GChem1 course. One reported study links the incorporation of LAs to an increased sense of belonging,^86^ a few studies in Computer Science explore the effect of POGIL on student belonging,^87,88^ and there is a qualitative study that discusses belonging in the context of PLTL.^89^ As far as the authors are aware, however, no previous studies have investigated the effects these high-impact practices have on the belonging felt by students holding various social identities. There is certainly a need for additional research in this area, but unfortunately the biases in our sense of belonging data precluded further investigation into this effect.

### Research Question 3b: Increased Persistence of Students to the First Organic Chemistry Course in the Series Was Mediated by Higher Grades Received in the Enhanced General Chemistry Course

Given the large body of literature which connects persistence to STEM course grades,^2,9,52,90^ we wanted to explore *post hoc* the relationship between course grade in GChem1 and persistence to OChem1. This felt especially relevant given that students in enhanced GChem1 received higher grades (mean 3.54 ± 0.68 SD) compared to students in standard GChem1 (mean 3.23 ± 0.86 SD, see Figure S5). Indeed, there was a statistically significant mediation effect of grades received in GChem1 on the association between type of GChem1 taken and persistence to OChem1. The average mediation effect was 0.06 for the first and second time point and 0.05 for the third time point (all *p* < 0.001). The strength of association between grades received in GChem1 and persistence to OChem1 was similar for both standard and enhanced GChem1 students (Figure S6, Tables S15). We do not believe the higher grades assigned in the enhanced course were a result of grade inflation given the similar performance of all students in OChem1 (see Research Question 1b). While the higher grades in enhanced GChem1 may be a reflection of better learning, it is also possible that this is a result of differences in course structure. As recent studies have shown, both grading scheme^91^ and assessment focus^92^ can have dramatic impacts on student grades.

## Conclusion

From our results, we can conclude that taking the enhanced version of general chemistry resulted in higher persistence to the first organic chemistry course in the series. This is in accordance with other studies.^20^ Our findings suggest that taking the enhanced course may result in higher persistence for women and NALA students as well. While the enhanced course is also linked to a higher sense of belonging (even when controlling for pre-belonging scores and prior academic preparation), we found that grade received in general chemistry was a significant predictor of persistence.

Several other studies have demonstrated a connection between persistence and grades in introductory STEM courses, and our work builds upon them by specifically looking at the impact of course reform efforts on this mechanism. Given that women and NALA students are more likely to receive a DFW in these courses, receiving a C or better has the potential to increase their chances of remaining in a STEM major.^3,53^ With that said, receiving anything lower than a B can also deter students from continuing in STEM.^2,93^ This is most likely due in part to the STEM-grading penalty,^94^ and it has been hypothesized that 2–4% more students would persist in STEM if the grade distributions in STEM courses were more similar to those in non-STEM courses.^52^

To be clear, we are not advocating for grade inflation. Students need a solid foundation in cross-cutting concepts in order to excel in subsequent courses, and we are doing a disservice to our students if we do not adequately prepare them. Instead, we are advocating for the use of evidence-based instructional practices such as POGIL, PLTL, and the use of LAs that improve learning for all students in all of our classrooms, and not just at the introductory level nor as a special case for select students. In other words, more STEM courses should be “enhanced”.

## Implications

This work underscores important themes that are emerging in Discipline-Based Education Research (DBER). High impact practices may not be serving all students in the same way, and as such, it is critical that we further disaggregate data in order to understand the effect these pedagogical methods have on students holding various identities.^95,96^ It is also imperative that we consider the intersectionality of certain identities, such as race/ethnicity and sex,^97^ given the known elevated risk of switching out of a STEM major for female NALA students.^2,3^

We also see our findings as yet another call for reflection on institutional practices.^92^ Our practices impact our students—from the types of assessments we use and the weight of those assessments,^91,98,99^ to the classroom culture we cultivate.^100^ It has been shown that students desire a major that reflects their values,^9^ and the importance of practices that affirm these values should not be underestimated.^101^ While modest increases in persistence can be had through the implementation of certain classroom practices, more is needed if we want to make STEM a welcoming place, especially for those whose identities and values may differ from those traditionally upheld by the discipline.

## Limitations and Disclosures

One major limitation of the study is how we define success (i.e., retention, grade received, and belonging). These definitions are based upon standards developed by privileged groups and therefore may not be representative of how our students view their success.^102^ Another major limitation of our study includes the use of SAT math and high school GPA as measures of prior preparation for general chemistry. We recognize that these are not the most accurate measures of preparedness, especially for certain subgroups.^103^ Additionally, we acknowledge the limitations associated with our grouping of Native Hawaiian/Pacific Islander, American Indian/Alaska Native, Latinx/Hispanic, and African American/Black students. How each of these groups experience racial oppression in the United States differs, and by grouping students together under the umbrella of NALA, we are ignoring differences in their histories in the United States and their lived experiences. This is also true for the classification of White and Asian/Asian American (WA), as there are many different ethnicities under the category of Asian, some of which are underrepresented in STEM. Furthermore, Asians and Asian Americans also face racial discrimination in the United States. Immigration policies such as the 1924 Immigration Act which was in place until 1952 and subsequent policies that largely only allow highly educated Asians into the US play a significant role in the seemingly large Asian representation in the US STEM workforce. Another limitation is that we were unable to account for socioeconomic, first-generation, and immigration status due to limited data available from the registrar. These classifications have been linked to persistence and by not considering the effects of our intervention on these groups, we are telling an incomplete story. We understand the importance of further disaggregating our data,^77,78,95^ but do not currently have the sample sizes required to do so. Finally, this study focuses only on on-sequence students but we recognize that results may differ for students who take the LS chemistry series off-sequence, and that the experiences had by this latter group of students warrants future investigation.

JCasey, SS, JCaram, AR, and AC were involved in the development of the enhanced series, and JCasey and AC were instructors for the enhanced courses. KS was not involved in the development nor the instruction of the enhanced series and therefore conducted all data analysis to reduce the potential for bias.

## Supporting Information Available

Details of Enhanced General Chemistry; Details of Standard General Chemistry; Belonging Surveys; Supplemental Figures and Tables; Supporting References (PDF) (DOCX)

## References

[ref1] Thorp H. H. Stop Passing the Buck on Intro Science. Science 2022, 378 (6616), 117–117. 10.1126/science.adf2231. 36205582

[ref2] Talking about Leaving Revisited: Persistence, Relocation, and Loss in Undergraduate STEM Education; Seymour E., Hunter A.-B., Eds.; Springer International Publishing: Cham, 2019. 10.1007/978-3-030-25304-2.

[ref3] Hatfield N.; Brown N. P.; Topaz C. M. Do Introductory STEM Courses Disproportionately Weed out Minoritized Students at Large, Public, Research-Intensive Universities? SocArXiv, February 14, 2022. 10.31235/osf.io/3gqps.

[ref4] Elliott R.; Strenta A. C.; Adair R.; Matier M.; Scott J. The Role of Ethnicity in Choosing and Leaving Science in Highly Selective Institutions. Res. High. Educ. 1996, 37 (6), 681–709. 10.1007/BF01792952.

[ref5] Asai D. J. Race Matters. Cell 2020, 181 (4), 754–757. 10.1016/j.cell.2020.03.044. 32413295

[ref6] Eagan K.; Hurtado S.; Figueroa T.; Hughes B. Examining STEM Pathways among Students Who Begin College at Four-Year Institutions; Commissioned Paper Prepared for the Committee on Barriers and Opportunities in Completing 2- and 4-Year STEM Degrees; National Academy of Sciences: Washington, DC, 2015, http://sites.nationalacademies.org/cs/groups/dbassesite/documents/webpage/dbasse_088834.pdf.

[ref7] Ong M.; Wright C.; Espinosa L.; Orfield G. Inside the Double Bind: A Synthesis of Empirical Research on Undergraduate and Graduate Women of Color in Science, Technology, Engineering, and Mathematics. Harv. Educ. Rev. 2011, 81 (2), 172–209. 10.17763/haer.81.2.t022245n7x4752v2.

[ref8] Chen X. STEM Attrition: College Students’ Paths into and out of STEM Fields; Statistical Analysis Report NCES 2014–001; National Center for Educational Statistics, 2013.

[ref9] Astorne-Figari C.; Speer J. D. Are Changes of Major Major Changes? The Roles of Grades, Gender, and Preferences in College Major Switching. Econ. Educ. Rev. 2019, 70, 75–93. 10.1016/j.econedurev.2019.03.005.

[ref10] Chang M. J.; Sharkness J.; Hurtado S.; Newman C. B. What Matters in College for Retaining Aspiring Scientists and Engineers from Underrepresented Racial Groups. J. Res. Sci. Teach. 2014, 51 (5), 555–580. 10.1002/tea.21146.

[ref11] George-Jackson C. E. STEM Switching: Examining Departures of Undergraduate Women in STEM Fields. J. Women Minor. Sci. Eng. 2011, 17 (2), 149–171. 10.1615/JWomenMinorScienEng.2011002912.

[ref12] Crabtree L. M.; Richardson S. C.; Lewis C. W. Gifted Gap, STEM Education, and Economic Immobility. J. Adv. Acad. 2019, 30 (2), 203–231. 10.1177/1932202X19829749.

[ref13] Hallett R. E.; Venegas K. M. Is Increased Access Enough? Advanced Placement Courses, Quality, and Success in Low-Income Urban Schools. J. Educ. Gift. 2011, 34 (3), 468–487. 10.1177/016235321103400305.

[ref14] Riegle-Crumb C.; King B.; Irizarry Y. Does STEM Stand Out? Examining Racial/Ethnic Gaps in Persistence Across Postsecondary Fields. Educ. Res. 2019, 48 (3), 133–144. 10.3102/0013189X19831006. PMC11244760 39005239

[ref15] White V.; Alexander J.; Verdell A. The Impact of Student Engagement, Institutional Environment, College Preparation, and Financial Support on the Persistence of Underrepresented Minority Student in Engineering at a Predominately White Institution. J. High Educ. Theory Pract. 2018, 10.33423/jhetp.v18i2.544.

[ref16] Davis L. P.; Museus S. D. What Is Deficit Thinking? An Analysis of Conceptualizations of Deficit Thinking and Implications for Scholarly Research. NCID Curr. 2019, 10.3998/currents.17387731.0001.110.

[ref17] Maton K. I.; Pollard S. A.; McDougall Weise T. V.; Hrabowski F. A. Meyerhoff Scholars Program: A Strengths-Based, Institution-Wide Approach to Increasing Diversity in Science, Technology, Engineering, and Mathematics: The Meyerhoff Scholars Program. Mt. Sinai J. Med. J. Transl. Pers. Med. 2012, 79 (5), 610–623. 10.1002/msj.21341. PMC3444508 22976367

[ref18] Sellami N.; Toven-Lindsey B.; Levis-Fitzgerald M.; Barber P. H.; Hasson T. A Unique and Scalable Model for Increasing Research Engagement, STEM Persistence, and Entry into Doctoral Programs. Life Sci. Educ. 2021, 20 (1), ar11 10.1187/cbe.20-09-0224. PMC8108502 33600221

[ref19] Freeman S.; Eddy S. L.; McDonough M.; Smith M. K.; Okoroafor N.; Jordt H.; Wenderoth M. P. Active Learning Increases Student Performance in Science, Engineering, and Mathematics. Proc. Natl. Acad. Sci. U. S. A. 2014, 111 (23), 8410–8415. 10.1073/pnas.1319030111. 24821756 PMC4060654

[ref20] Theobald E. J.; Hill M. J.; Tran E.; Agrawal S.; Arroyo E. N.; Behling S.; Chambwe N.; Cintrón D. L.; Cooper J. D.; Dunster G.; Grummer J. A.; Hennessey K.; Hsiao J.; Iranon N.; Jones L.; Jordt H.; Keller M.; Lacey M. E.; Littlefield C. E.; Lowe A.; Newman S.; Okolo V.; Olroyd S.; Peecook B. R.; Pickett S. B.; Slager D. L.; Caviedes-Solis I. W.; Stanchak K. E.; Sundaravardan V.; Valdebenito C.; Williams C. R.; Zinsli K.; Freeman S. Active Learning Narrows Achievement Gaps for Underrepresented Students in Undergraduate Science, Technology, Engineering, and Math. Proc. Natl. Acad. Sci. U. S. A. 2020, 117 (12), 6476–6483. 10.1073/pnas.1916903117. 32152114 PMC7104254

[ref21] Haak D. C.; HilleRisLambers J.; Pitre E.; Freeman S. Increased Structure and Active Learning Reduce the Achievement Gap in Introductory Biology. Science 2011, 332 (6034), 1213–1216. 10.1126/science.1204820. 21636776

[ref22] Eddy S. L.; Hogan K. A. Getting Under the Hood: How and for Whom Does Increasing Course Structure Work?. Life Sci. Educ. 2014, 13 (3), 453–468. 10.1187/cbe.14-03-0050. PMC4152207 25185229

[ref23] Nardo J. E.; Chapman N. C.; Shi E. Y.; Wieman C.; Salehi S. Perspectives on Active Learning: Challenges for Equitable Active Learning Implementation. J. Chem. Educ. 2022, 99 (4), 1691–1699. 10.1021/acs.jchemed.1c01233.

[ref24] Chapter 1. POGIL: An Overview. In Process Oriented Guided Inquiry Learning (POGIL); Moog R. S., Spencer J. N., Eds.; ACS Symposium Series; American Chemical Society: Washington, DC, 2008. 10.1021/bk-2008-0994.

[ref25] Farrell J. J.; Moog R. S.; Spencer J. N. A Guided-Inquiry General Chemistry Course. J. Chem. Educ. 1999, 76 (4), 570 10.1021/ed076p570.

[ref26] Rodriguez J.-M. G.; Hunter K. H.; Scharlott L. J.; Becker N. M. A Review of Research on Process Oriented Guided Inquiry Learning: Implications for Research and Practice. J. Chem. Educ. 2020, 97 (10), 3506–3520. 10.1021/acs.jchemed.0c00355.

[ref27] Chapter 12. POGIL in Chemistry Courses at a Large Urban University: A Case Study. In Process Oriented Guided Inquiry Learning (POGIL); Moog R. S., Spencer J. N., Eds.; ACS Symposium Series; American Chemical Society: Washington, DC, 2008, 10.1021/bk-2008-09.

[ref30] Chapter 6. POGIL Implementation in Large Classes: Strategies for Planning, Teaching, and Management. In Process Oriented Guided Inquiry Learning (POGIL); Moog R. S., Spencer J. N., Eds.; ACS Symposium Series; American Chemical Society: Washington, DC, 2008. 10.1021/bk-2008-0994.

[ref28] Hein S. M. Positive Impacts Using POGIL in Organic Chemistry. J. Chem. Educ. 2012, 89 (7), 860–864. 10.1021/ed100217v.

[ref29] Chapter 19. A Multi-Institutional Assessment of the Use of POGIL in Organic Chemistry. In Process Oriented Guided Inquiry Learning (POGIL); Moog R. S., Spencer J. N., Eds.; ACS Symposium Series; American Chemical Society: Washington, DC, 2008. 10.1021/bk-2008-0994.

[ref31] Gosser D. K. Peer-Led Team Learning: A Guidebook; Prentice Hall: Upper Saddle River, NJ, 2001.

[ref32] Raker J. R.; Dood A. J.; Srinivasan S.; Murphy K. L. Pedagogies of Engagement Use in Postsecondary Chemistry Education in the United States: Results from a National Survey. Chem. Educ. Res. Pract. 2021, 22 (1), 30–42. 10.1039/D0RP00125B.

[ref33] Wilson S. B.; Varma-Nelson P. Small Groups, Significant Impact: A Review of Peer-Led Team Learning Research with Implications for STEM Education Researchers and Faculty. J. Chem. Educ. 2016, 93 (10), 1686–1702. 10.1021/acs.jchemed.5b00862.

[ref34] Cracolice M. S. Vygotsky’s Zone of Proximal Development: A Theory Base for Peer-Led Team Learning. Progress. PLTL Proj. Newsl. 2000, 1 ( (2), ). http://pltlis.org/wp-content/uploads/2012/10/PLTL-and-Vygotsky-Vygotsky-ZPD-Cracolice.pdf.

[ref35] Lewis S. E.; Lewis J. E. Departing from Lectures: An Evaluation of a Peer-Led Guided Inquiry Alternative. J. Chem. Educ. 2005, 82 (1), 135 10.1021/ed082p135.

[ref36] Golde M. F.; McCreary C. L.; Koeske R. Peer Instruction in the General Chemistry Laboratory: Assessment of Student Learning. J. Chem. Educ. 2006, 83 (5), 804 10.1021/ed083p804.

[ref37] Wamser C. C. Peer-Led Team Learning in Organic Chemistry: Effects on Student Performance, Success, and Persistence in the Course. J. Chem. Educ. 2006, 83 (10), 1562 10.1021/ed083p1562.

[ref38] Otero V.; Pollock S.; Finkelstein N. A Physics Department’s Role in Preparing Physics Teachers: The Colorado Learning Assistant Model. Am. J. Phys. 2010, 78 (11), 1218 10.1119/1.3471291.

[ref39] Barrasso A. P.; Spilios K. E. A Scoping Review of Literature Assessing the Impact of the Learning Assistant Model. Int. J. STEM Educ. 2021, 8 (1), 12 10.1186/s40594-020-00267-8.

[ref40] Talbot R. M.; Hartley L. M.; Matzetta K.; Wee B. S. Transforming Undergraduate Science Education With Learning Assistants: Student Satisfaction in Large-Enrollment Courses. J. Coll. Sci. Teach. 2015, 44 (5), 24–30.

[ref41] Alzen J. L.; Langdon L. S.; Otero V. K. A Logistic Regression Investigation of the Relationship between the Learning Assistant Model and Failure Rates in Introductory STEM Courses. Int. J. STEM Educ. 2018, 5 (1), 56 10.1186/s40594-018-0152-1. 30631745 PMC6310447

[ref104] Rempel B. P.; Dirks M. B.; McGinitie E. G. Two-Stage Testing Reduces Student-Perceived Exam Anxiety in Introductory Chemistry. J. Chem. Educ. 2021, 98, 2527 10.1021/acs.jchemed.1c00219.

[ref42] Van Dusen B.; Nissen J. Associations between Learning Assistants, Passing Introductory Physics, and Equity: A Quantitative Critical Race Theory Investigation. Phys. Rev. Phys. Educ. Res. 2020, 16 (1), 010117 10.1103/PhysRevPhysEducRes.16.010117.

[ref43] Eberlein T.; Kampmeier J.; Minderhout V.; Moog R. S.; Platt T.; Varma-Nelson P.; White H. B. Pedagogies of Engagement in Science: A Comparison of PBL, POGIL, and PLTL. Biochem. Mol. Biol. Educ. 2008, 36 (4), 262–273. 10.1002/bmb.20204. 19381266 PMC2665262

[ref44] Niemiec C. P.; Ryan R. M. Autonomy, Competence, and Relatedness in the Classroom: Applying Self-Determination Theory to Educational Practice. Theory Res. Educ. 2009, 7 (2), 133–144. 10.1177/1477878509104318.

[ref45] Ryan R. M.; Niemiec C. P. Self-Determination Theory in Schools of Education: Can an Empirically Supported Framework Also Be Critical and Liberating?. Theory Res. Educ. 2009, 7 (2), 263–272. 10.1177/1477878509104331.

[ref46] Shell D. F.; Brooks D. W.; Trainin G.; Wilson K. M.; Kauffman D. F.; Herr L. M. Chapter 8: Supporting Motivation. In The Unified Learning Model: How Motivational, Cognitive, and Neurobiological Sciences Inform Best Teaching Practices; Springer: Dordrecht, 2010; pp 65–85.

[ref47] Jeno L. M.; Raaheim A.; Kristensen S. M.; Kristensen K. D.; Hole T. N.; Haugland M. J.; Mæland S. The Relative Effect of Team-Based Learning on Motivation and Learning: A Self-Determination Theory Perspective. Life Sci. Educ. 2017, 16 (4), ar59 10.1187/cbe.17-03-0055. PMC5749961 29146665

[ref48] Tinto V. Leaving College: Rethinking the Causes and Cures of Student Attrition, 2nd ed.; University of Chicago Press: Chicago, 1993.

[ref49] Hurtado S.; Carter D. F. Effects of College Transition and Perceptions of the Campus Racial Climate on Latino College Students’ Sense of Belonging. Sociol. Educ. 1997, 70 (4), 324 10.2307/2673270.

[ref50] Hausmann L. R. M.; Schofield J. W.; Woods R. L. Sense of Belonging as a Predictor of Intentions to Persist Among African American and White First-Year College Students. Res. High. Educ. 2007, 48 (7), 803–839. 10.1007/s11162-007-9052-9.

[ref51] Fink A.; Frey R. F.; Solomon E. D. Belonging in General Chemistry Predicts First-Year Undergraduates’ Performance and Attrition. Chem. Educ. Res. Pract. 2020, 21 (4), 1042–1062. 10.1039/D0RP00053A.

[ref52] Rask K. Attrition in STEM Fields at a Liberal Arts College: The Importance of Grades and Pre-Collegiate Preferences. Econ. Educ. Rev. 2010, 29, 892 10.1016/j.econedurev.2010.06.013.

[ref53] Harris R. B.; Mack M. R.; Bryant J.; Theobald E. J.; Freeman S. Reducing Achievement Gaps in Undergraduate General Chemistry Could Lift Underrepresented Students into a “Hyperpersistent Zone.. Sci. Adv. 2020, 6 (24), eaaz5687 10.1126/sciadv.aaz5687. 32577510 PMC7286681

[ref54] Kennepohl D.; Guay M.; Thomas V. Using an Online, Self-Diagnostic Test for Introductory General Chemistry at an Open University. J. Chem. Educ. 2010, 87 (11), 1273–1277. 10.1021/ed900031p.

[ref55] Women, Minorities, and Persons with Disabilities in Science and Engineering: 2021. Special Report; National Center for Science and Engineering Statistics; National Science Foundation: Alexandria, VA, 2021. https://ncses.nsf.gov/wmpd.

[ref56] Tai R. H.; Sadler P. M.; Loehr J. F. Factors Influencing Success in Introductory College Chemistry. J. Res. Sci. Teach. 2005, 42 (9), 987–1012. 10.1002/tea.20082.

[ref57] Xu Y. J. Career Outcomes of STEM and Non-STEM College Graduates: Persistence in Majored-Field and Influential Factors in Career Choices. Res. High Educ 2013, 54, 349 10.1007/s11162-012-9275-2.

[ref58] Edwards J. D.; Barthelemy R. S.; Frey R. F. Relationship between Course-Level Social Belonging (Sense of Belonging and Belonging Uncertainty) and Academic Performance in General Chemistry 1. J. Chem. Educ. 2022, 99 (1), 71–82. 10.1021/acs.jchemed.1c00405.

[ref59] R Core Team. R: A Language and Environment for Statistical Computing, 2019. https://www.R-project.org/.

[ref60] Wickham H.; Chang W. An Implementation of the Grammar of Graphics, 2014, https://cran.r-project.org/web/packages/ggplot2/index.html.

[ref61] Wickham H.; Girlich M. Tidyr: Tidy Messy Data, 2022. https://cran.r-project.org/web/packages/tidyr/index.html.

[ref62] Lüdecke D. SjPlot: Data Visualization for Statistics in Social Science, 2022. https://cran.r-project.org/web/packages/sjPlot/index.html.

[ref63] Sjoberg D. D.; Curry M.; Larmarange J.; Lavery J.; Whiting K.; Zabor E. C. Gtsummary: Presentation-Ready Data Summary and Analytic Result Tables, 2022. https://cran.r-project.org/web/packages/gtsummary/index.html.

[ref64] Pedersen T. L. Patchwork: The Composer of Plots, 2020, https://cloud.r-project.org/web/packages/patchwork/patchwork.pdf.

[ref65] Rosseel Y. Lavaan: An *R* Package for Structural Equation Modeling. J. Stat. Softw. 2012, 10.18637/jss.v048.i02.

[ref66] Tingley D.; Yamamoto T.; Hirose K.; Keele L.; Imai K. Mediation: R Package for Causal Mediation Analysis. J. Stat. Softw. 2014, 59 (5), 1–38. 10.18637/jss.v059.i05.26917999

[ref67] Knekta E.; Runyon C.; Eddy S. One Size Doesn’t Fit All: Using Factor Analysis to Gather Validity Evidence When Using Surveys in Your Research. Life Sci. Educ. 2019, 18, 17 10.1187/cbe.18-04-0064. PMC6757227 30821600

[ref68] Imai K.; Keele L.; Tingley D. A General Approach to Causal Mediation Analysis. Psychol. Methods 2010, 15 (4), 309–334. 10.1037/a0020761. 20954780

[ref69] Schnoebelen C.; Towns M. H.; Chmielewski J.; Hrycyna C. A. Design and Evaluation of a One-Semester General Chemistry Course for Undergraduate Life Science Majors. J. Chem. Educ. 2018, 95 (5), 734–740. 10.1021/acs.jchemed.7b00869.

[ref70] Ege S. N.; Coppola B. P.; Lawton R. G. The University of Michigan Undergraduate Chemistry Curriculum 1. Philosophy, Curriculum, and the Nature of Change. J. Chem. Educ. 1997, 74 (1), 74 10.1021/ed074p74.

[ref71] Reingold I. D. Bioorganic First: A New Model for the College Chemistry W Curriculum. J. Chem. Educ. 2001, 78, 869–871. 10.1021/ed078p869.

[ref72] Billing D. Teaching for Transfer of Core/Key Skills in Higher Education: Cognitive Skills. High. Educ. 2007, 53 (4), 483–516. 10.1007/s10734-005-5628-5.

[ref73] Snyder J. J.; Sloane J. D.; Dunk R. D. P.; Wiles J. R. Peer-Led Team Learning Helps Minority Students Succeed. PLOS Biol. 2016, 14 (3), e1002398 10.1371/journal.pbio.1002398. 26959826 PMC4784972

[ref74] Sloane J. D.; Dunk R. D. P.; Snyder J. J.; Winterton C. I.; Wiles J. R. Peer-Led Team Learning Is Associated with an Increased Retention Rate for STEM Majors from Marginalized Groups. In 13th Annual ANBT Biology Education Research Symposium; Atlanta, GA, p 9.

[ref75] Gafney L.; Varma-Nelson P. Chapter 7: Impact on Minority Students and Women. In Peer-Led Team Learning: Evaluation, Dissemination, and Institutionalization of a College Level Initiative; Spring Science + Business Media B.V., 2008; pp 87–95.

[ref76] Bancroft S. F.; Fowler S. R.; Jalaeian M.; Patterson K. Leveling the Field: Flipped Instruction as a Tool for Promoting Equity in General Chemistry. J. Chem. Educ. 2020, 97 (1), 36–47. 10.1021/acs.jchemed.9b00381.

[ref77] Bancroft S. F.; Jalaeian M.; John S. R. Systematic Review of Flipped Instruction in Undergraduate Chemistry Lectures (2007–2019): Facilitation, Independent Practice, Accountability, and Measure Type Matter. J. Chem. Educ. 2021, 98 (7), 2143–2155. 10.1021/acs.jchemed.0c01327.

[ref78] Almeida K. H. Disaggregated General Chemistry Grades Reveal Differential Success among BIPOC Students in Partial Flipped Team Learning Classrooms. J. Chem. Educ. 2022, 99 (1), 259–267. 10.1021/acs.jchemed.1c00401.

[ref79] Lewis K. L.; Stout J. G.; Finkelstein N. D.; Pollock S. J.; Miyake A.; Cohen G. L.; Ito T. A. Fitting in to Move Forward: Belonging, Gender, and Persistence in the Physical Sciences, Technology, Engineering, and Mathematics (PSTEM). Psychol. Women Q. 2017, 41 (4), 420–436. 10.1177/0361684317720186.

[ref80] Dortch D.; Patel C. Black Undergraduate Women and Their Sense of Belonging in STEM at Predominantly White Institutions. NASPA J. Women High. Educ. 2017, 10 (2), 202–215. 10.1080/19407882.2017.1331854.

[ref81] Johnson D. R. Women of Color in Science, Technology, Engineering, and Mathematics (STEM). New Dir. Institutional Res. 2011, 2011 (152), 75–85. 10.1002/ir.410.

[ref82] Rainey K.; Dancy M.; Mickelson R.; Stearns E.; Moller S. Race and Gender Differences in How Sense of Belonging Influences Decisions to Major in STEM. Int. J. STEM Educ. 2018, 5 (1), 10 10.1186/s40594-018-0115-6. 30631700 PMC6310405

[ref83] Freeman T. M.; Anderman L. H.; Jensen J. M. Sense of Belonging in College Freshmen at the Classroom and Campus Levels. Journal of Experimental Education 2007, 75 (3), 203–220. 10.3200/JEXE.75.3.203-220.

[ref84] Walton G. M.; Cohen G. L. A Brief Social-Belonging Intervention Improves Academic and Health Outcomes of Minority Students. Science 2011, 331 (6023), 1447–1451. 10.1126/science.1198364. 21415354

[ref85] Walton G. M.; Logel C.; Peach J. M.; Spencer S. J.; Zanna M. P. Two Brief Interventions to Mitigate a “Chilly Climate” Transform Women’s Experience, Relationships, and Achievement in Engineering. J. Educ. Psychol. 2015, 107 (2), 468–485. 10.1037/a0037461.

[ref86] Clements T. P.; Friedman K. L.; Johnson H. J.; Meier C. J.; Watkins J.; Brockman A. J.; Brame C. J. “It Made Me Feel like a Bigger Part of the STEM Community”: Incorporation of Learning Assistants Enhances Students’ Sense of Belonging in a Large Introductory Biology Course. Life Sci. Educ. 2022, 21 (2), ar26 10.1187/cbe.21-09-0287. PMC9508922 35412327

[ref87] Moudgalya S. K.; Mayfield C.; Yadav A.; Hu H. H.; Kussmaul C. Measuring Students’ Sense of Belonging in Introductory CS Courses. In Proceedings of the 52nd ACM Technical Symposium on Computer Science Education; ACM: Virtual Event, USA, 2021; pp 445–451. 10.1145/3408877.3432425.

[ref88] Mayfield C.; Moudgalya S. K.; Yadav A.; Kussmaul C.; Hu H. H. POGIL in CS1: Evidence for Student Learning and Belonging. In Proceedings of the 53rd ACM Technical Symposium on Computer Science Education; ACM: Providence, RI, 2022; pp 439–445, 10.1145/3478431.349929.

[ref89] Villa E. Q. Minority Voices: Interrupting the Social Environment to Retain Undergraduates in Computing. ACM Inroads 2018, 9 (3), 31–33. 10.1145/3239257.

[ref90] Dika S. L.; D’Amico M. M. Early Experiences and Integration in the Persistence of First-Generation College Students in STEM and Non-STEM Majors. J. Res. Sci. Teach. 2016, 53 (3), 368–383. 10.1002/tea.21301.

[ref91] Tashiro J.; Talanquer V. Exploring Inequities in a Traditional and a Reformed General Chemistry Course. J. Chem. Educ. 2021, 98 (12), 3680–3692. 10.1021/acs.jchemed.1c00821.

[ref92] Ralph V. R.; Scharlott L. J.; Schafer A. G. L.; Deshaye M. Y.; Becker N. M.; Stowe R. L. Advancing Equity in STEM: The Impact Assessment Design Has on Who Succeeds in Undergraduate Introductory Chemistry. JACS Au 2022, 2 (8), 1869–1880. 10.1021/jacsau.2c00221. 36032534 PMC9400050

[ref93] Main J. B.; Mumford K. J.; Ohland M. W. Examining the Influence of Engineering Students’ Course Grades on Major Choice and Major Switching Behavior. Int. J. Eng. Educ. 2015, 31 (6), 1468–1475.

[ref94] Witteveen D.; Attewell P. The STEM Grading Penalty: An Alternative to the “Leaky Pipeline” Hypothesis. Sci. Educ. 2020, 104 (4), 714–735. 10.1002/sce.21580.

[ref95] Collins J. S.; Olesik S. V. The Important Role of Chemistry Department Chairs and Recommendations for Actions They Can Enact to Advance Black Student Success. J. Chem. Educ. 2021, 98 (7), 2209–2220. 10.1021/acs.jchemed.0c01329.

[ref96] Arnaud C. H. Weeding out Inequity in Undergraduate Chemistry Classes. C&EN Glob. Enterp. 2020, 98 (34), 34–37. 10.1021/cen-09834-cover3.

[ref97] Cochran G. L.; Boveda M.; Prescod-Weinstein C. Intersectionality in STEM Education Research. In Handbook of Research on STEM Education; Routledge, 2020; pp 257–266.

[ref98] Simmons A. B.; Heckler A. F. Grades, Grade Component Weighting, and Demographic Disparities in Introductory Physics. Phys. Rev. Phys. Educ. Res. 2020, 16 (2), 020125 10.1103/PhysRevPhysEducRes.16.020125.

[ref99] Shah L.; Fatima A.; Syed A.; Glasser E. Investigating the Impact of Assessment Practices on the Performance of Students Perceived to Be at Risk of Failure in Second-Semester General Chemistry. J. Chem. Educ. 2022, 99 (1), 14–24. 10.1021/acs.jchemed.0c01463.

[ref100] Canning E. A.; Muenks K.; Green D. J.; Murphy M. C. STEM Faculty Who Believe Ability Is Fixed Have Larger Racial Achievement Gaps and Inspire Less Student Motivation in Their Classes. Sci. Adv. 2019, 5 (2), eaau4734 10.1126/sciadv.aau4734. 30793027 PMC6377274

[ref101] Harackiewicz J. M.; Canning E. A.; Tibbetts Y.; Giffen C. J.; Blair S. S.; Rouse D. I.; Hyde J. S. Closing the Social Class Achievement Gap for First-Generation Students in Undergraduate Biology. J. Educ. Psychol. 2014, 106 (2), 375–389. 10.1037/a0034679. 25049437 PMC4103196

[ref102] Weatherton M.; Schussler E. E. Success for All? A Call to Re-Examine How Student Success Is Defined in Higher Education. Life Sci. Educ. 2021, 20 (1), es3 10.1187/cbe.20-09-0223. PMC8108506 33635125

[ref103] Kurlaender M.; Cohen. Predicting College Success: How Do Different High School Assessments Measure Up?; Policy Analysis for California Education, 2019; p 35.

