# Supplementary material for: Participation in a High-Structure General Chemistry Course Increases Student Sense of Belonging and Persistence to Organic Chemistry
Source: J Chem Educ. 2023 Jul 12;100(8):2860–72. doi: 10.1021/acs.jchemed.2c01253 (PMC10413951; doi:10.1021/acs.jchemed.2c01253)
Supplement: Supplementary file 1 — ed2c01253_si_001.pdf [file ed2c01253_si_001.pdf]

Supporting Information

Participation in High-Structure General Chemistry Course Increases Student Sense of Belonging and Persistence to Organic Chemistry

Jennifer R. Casey[1]*, K. Supriya[2], Shanna Shaked[2], Justin R. Caram[1], Arlene Russell[1], Albert J. Courey[1]

[1] Department of Chemistry and Biochemistry, UCLA, Los Angeles, California 90095, United States

[2] Center for Education, Innovation, and Learning in the Sciences, UCLA, Los Angeles, California 90095, United States

*E-mail: jrcasey@chem.ucla.edu

Details of Enhanced General Chemistry.

Lecture: Lecture met three times a week for 50-minutes. Some active learning strategies were used during lecture, such as student polling and think-pair-share. The primary focus was on the presentation of material and working through practice problems, with approximately 30% of lecture time allocated for student discussion. Final grades were assigned based on a variety of assessments, including high-stakes exams (45%) and discussion group worksheets (12%).

Discussion: The weekly mandatory discussion sections were 110-minutes and taught by graduate Teaching Assistants (TA). Students were randomly assigned to teams of three or four. These assignments were generally permanent, unless either a student dropped the course and so teams had to be rearranged to maintain an adequate number of teammates, or if a student specifically requested to switch teams due to personal conflicts. Each team was also assigned a permanent Learning Assistant (LA), whose responsibility was to facilitate group work. Ideally the LA allowed team members to work independently and would only intervene to encourage dialogue when necessary. Each team member was assigned a role (Manager, Presenter, Recorder, or Reflector), and these roles changed weekly. Teams were given a structured worksheet that was designed by the instructor, but was focused on the learning cycle (model exploration, concept invention, and application). The Recorder was responsible for turning in the team's final version of the worksheet to the TA before the following discussion section. After receiving feedback on their worksheet, teams were allowed to correct and resubmit the worksheet in order to receive a higher score.

Details of Standard General Chemistry. The standard general chemistry courses investigated in this study include those taught during Fall 2017, Fall 2018, Fall 2019, and Fall 2020. During this timeframe, three different instructors taught the course and therefore there was wide variability in the lecture and discussion structure.

Lecture: Lecture met three times a week for 50-minutes. Active learning strategies were sometimes used, specifically student polling. The primary focus was usually on the presentation of material and working through practice problems, with less time allocated for student discussion. Final grades were assigned based on a variety of assessments, including high-stakes exams (60%-75%) and participation (10-15%).

Supporting Information

Discussion: Weekly discussion sections taught by graduate Teaching Assistants were considered mandatory, but the extent of active learning used in discussion varied greatly. While no discussion sections used POGIL, some did utilize group work and Learning Assistants (specifically S F17 I1, S F18 I3, and S F19 I3 in Table 2 of the manuscript). If discussion worksheets were used, they were not collected but participation points were given to students who actively worked on the assigned materials.

Belonging Surveys:

Pre-Survey
Please indicate the level to which you agree with the following statement in relation to your previous general chemistry course(s).

1. I felt like I fit.
2. I felt comfortable with my peers and classmates.
3. I felt comfortable with my instructor(s).
4. Setting aside my performance, I felt like I belonged.
5. I felt uncertain about my belonging.
6. If I didn't perform, I felt like maybe I didn't belong.

Post-Survey
Please indicate the level to which you agree with the following statement in relation to your current general chemistry course.

1. I feel like I fit.
2. I feel comfortable with my peers and classmates.
3. I feel comfortable with my instructor(s).
4. Setting aside my performance, I feel like I belong.
5. I feel uncertain about my belonging.
6. If I don't perform, I feel like maybe I don't belong.

Supplemental Figures and Tables

Table S1. Demographic data for students included in this study.

	**Data for students with SAT math scores**			**Data for students with high school GPA but not SAT math scores**		
	**All terms**	**Fall 2020 term**		**All terms**	**Fall 2020 term**	
**Characteristic**	**Standard GChem1 (N= 3,100)**	**Standard GChem1 (N= 749)**	**Enhanced GChem1 (N= 176)**	**Standard GChem1 (N= 4,027)**	**Standard GChem1 (N= 922)**	**Enhanced GChem1 (N= 233)**
Average SAT Math Score	707 ± 82 SD	712 ± 81 SD	699 ± 81 SD	-	-	-
Average High School GPA	4.47 ± 0.25 SD	4.50 ± 0.24 SD	4.48 ± 0.21 SD	4.48 ± 0.25 SD	4.51 ± 0.24 SD	4.49 ± 0.22 SD
Score on a chemistry diagnostic exam taken prior to GChem1 course	-	3.27 ± 0.53 SD (N= 531)	3.00 ± 0.60 SD (N= 188)	-	-	-
% of students that either took AP chemistry with a grade ≥ 4 or community college chemistry with a grade ≥ B	-	40.31% (N= 531)	24.47% (N= 176)	-	-	-
**Sex**						
Female	2,120 (68.4%)	511 (68.2%)	126 (71.6%)	2,782 (69.1%)	634 (68.8%)	171 (73.4%)
Male	976 (31.5%)	238 (31.8%)	50 (28.4%)	1,236 (30.7%)	285 (30.9%)	61 (26.2%)
Non-binary/Unknown	0.1%	0%	0%	0.2%	0.3%	0.4%
**Race/ethnicity**						
WA (White, Asian/Asian American)	2,301 (74.2%)	549 (73.3%)	128 (72.7%)	3,101 (77.0%)	684 (74.2%)	175 (75.1%)
NALA (Native Hawaiian/Pacific Islander, American Indian/Native American, Latinx/Hispanic, African American/Black)	739 (23.8%)	182 (24.3%)	39 (22.2%)	838 (20.8%)	213 (23.1%)	44 (18.9%)
Unknown	60 (1.9%)	18 (2.4%)	9 (5.1%)	88 (2.2%)	25 (2.7%)	14 (6.0%)

Table S2. Logistic regression results for student persistence to first organic chemistry course in the series at three different time points. SAT math scores and high school GPA were scaled to a mean of 0 and standard deviation of 1.

	**First time point**			**Second time point**			**Third time point**		
	**log(OR)**	**95% CI**	**p-value**	**log(OR)**	**95% CI**	**p-value**	**log(OR)**	**95% CI**	**p-value**
(Intercept)	0.45	0.13, 0.77	0.006	1.5	1.1, 1.9	<0.001	2.0	1.6, 2.5	<0.001
**GChem1 Course type**									
Standard	—	—		—	—		—	—	
Enhanced	0.44	-0.02, 0.90	0.061	0.59	0.05, 1.2	0.034	0.60	-0.01, 1.1	0.055
**Instructor**									
I3	—	—		—	—		—	—	
I1	-0.49	-0.90, -0.09	0.017	-0.47	-0.94, 0.00	0.052	-0.54	-1.1, -0.01	0.048
I2	-0.20	-0.46, 0.05	0.11	-0.26	-0.56, 0.03	0.084	-0.15	-0.49, 0.19	0.4
**Term when GChem1 was taken**									
17F	—	—		—	—		—	—	
18F	-0.54	-0.79, -0.30	<0.001	-0.36	-0.65, -0.08	0.012	-0.28	-0.62, 0.05	0.10
19F	-0.14	-0.38, 0.10	0.3	-0.15	-0.44, 0.14	0.3	-0.30	-0.64, 0.04	0.086
20F	-0.11	-0.36, 0.13	0.4	-0.20	-0.48, 0.09	0.2	-0.33	0.67, 0.01	0.057
**z-SAT Math score**	0.03	-0.05, 0.10	0.5	0.24	0.16, 0.32	<0.001	0.33	0.24, 0.42	<0.001
**z-High school GPA**	0.24	0.16, 0.31	<0.001	0.35	0.27, 0.43	<0.001	0.41	0.32, 0.51	<0.001
AIC	4,446			3,590			2,877		
OR = Odds Ratio, CI = Confidence Interval									

Supporting Information

Figure S1. Predicted values of % student persistence to the first-quarter organic chemistry course at different time points based on logistic regression models with GChem1 course as the predictor. The models controlled for term, instructor, and high-school GPA.

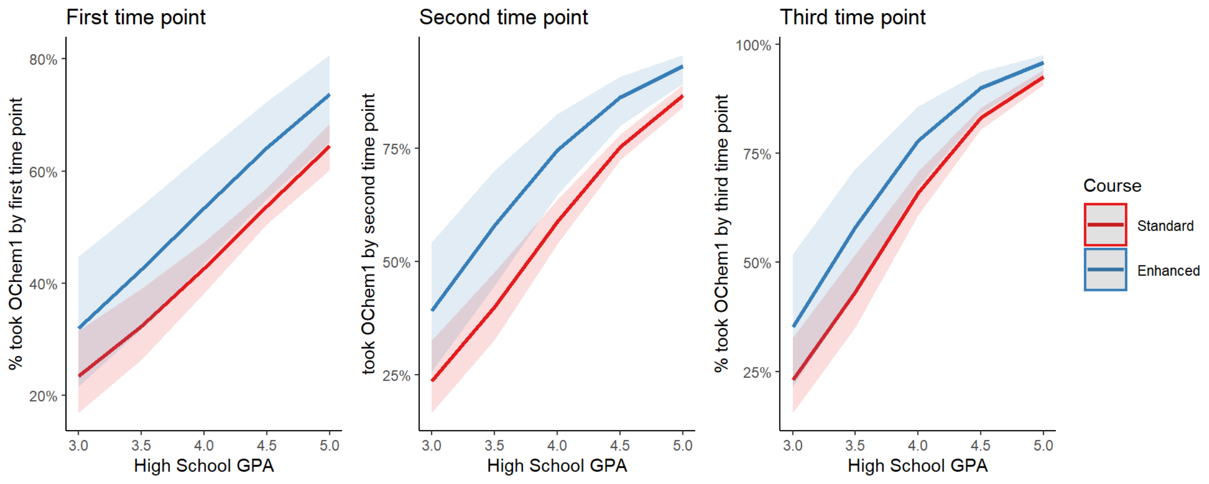

Table S3. Logistic regression results for student persistence to first organic chemistry course in the series at three different time points for the larger data set. High school GPA was scaled to a mean of 0 and standard deviation of 1.

	**First time point**			**Second time point**			**Third time point**		
	**log(OR)**	**95% CI**	**p-value**	**log(OR)**	**95% CI**	**p-value**	**log(OR)**	**95% CI**	**p-value**
(Intercept)	0.30	0.02, 0.58	0.033	1.4	1.0, 1.7	<0.001	1.9	1.6, 2.3	<0.001
**GChem1 Course type**									
Standard	—	—		—	—		—	—	
Enhanced	0.43	0.03, 0.84	0.035	0.73	0.25, 1.2	0.003	0.60	0.06, 1.1	0.031
**Instructor**									
I3	—	—		—	—		—	—	
I1	-0.33	-0.69, 0.02	0.063	-0.41	-0.81, 0.00	0.048	-0.48	-0.95, -0.02	0.043
I2	-0.09	-0.31, 0.14	0.5	-0.11	-0.37, 0.14	0.4	-0.13	-0.43, 0.17	0.4
**Term when GChem1 was taken**									
Fall 2017	—	—		—	—		—	—	
Fall 2018	-0.48	-0.69, -0.27	<0.001	-0.33	-0.58, -0.09	0.007	-0.27	-0.56, 0.01	0.063
Fall 2019	-0.07	-0.28, 0.14	0.5	-0.09	-0.34, 0.16	0.5	-0.14	-0.44, 0.14	0.3
Fall 2020	-0.08	-0.29, 0.13	0.4	-0.16	-0.41, 0.09	0.2	-0.25	-0.54, 0.04	0.095
**z-High school GPA**	0.22	0.16, 0.28	<0.001	0.37	0.30, 0.44	<0.001	0.46	0.38, 0.53	<0.001
AIC	5,732			4,640			3,749		
OR = Odds Ratio, CI = Confidence Interval									

Table S4. Linear regression results for student grade in first organic chemistry course in the series. SAT math scores and high school GPA were scaled to a mean of 0 and standard deviation of 1.

	**Beta**	**95% CI**	**p-value**
(Intercept)	3.1	2.9, 3.3	<0.001
**GChem1 Course type**			
Standard	—	—	
Enhanced	0.07	-0.07, 0.21	0.4
**Term when OChem1 was taken**			
Summer 2021	—	—	
Fall 2021	0.12	-0.12, 0.36	0.3
Spring 2021	0.48	0.26, 0.70	<0.001
Winter 2022	0.08	-0.21, 0.36	0.6
**z-SAT Math Score**	0.35	0.29, 0.41	<0.001
**z-High School GPA**	0.04	-0.02, 0.10	0.2
R^2	0.216		
AIC	1,772		

CI = Confidence Interval

Supporting Information

Figure S2. Predicted grades in the first-quarter organic chemistry course based on linear regression models with GChem1 course as the predictor. The models controlled for term in which organic chemistry was taken and high school GPA.

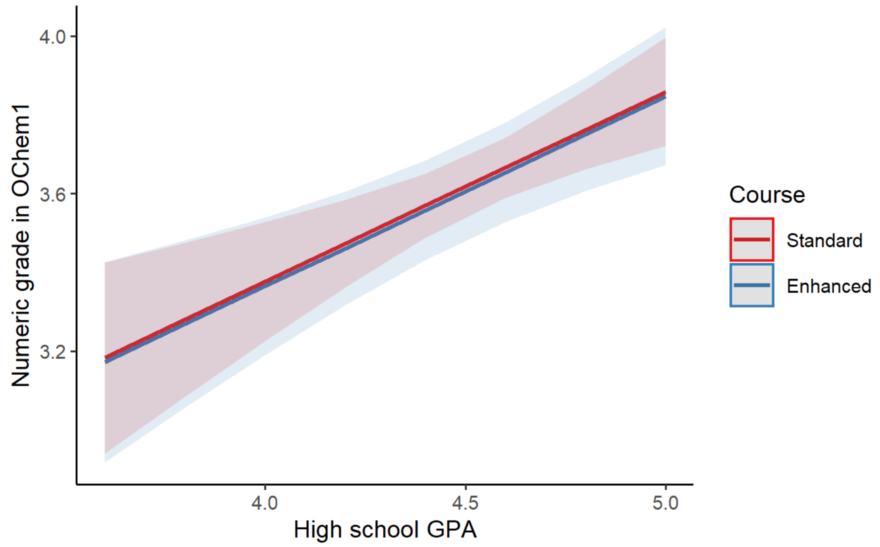

Table S5. Linear regression results for student grade in first organic chemistry course in the series using the larger dataset. High school GPA was scaled to a mean of 0 and standard deviation of 1.

	**Beta**	**95% CI**	**p-value**
(Intercept)	3.1	2.9, 3.3	<0.001
**GChem1 Course type**			
Standard	—	—	
Enhanced	-0.01	-0.14, 0.12	0.9
**Term when OChem1 was taken**			
Summer 2021	—	—	
Fall 2021	0.24	0.02, 0.47	0.034
Spring 2021	0.56	0.35, 0.77	<0.001
Winter 2022	0.11	-0.17, 0.38	0.5
**z-High School GPA**	0.10	0.05, 0.16	<0.001
R^2	0.069		
AIC	2,341		

CI = Confidence Interval

Supporting Information

Figure S3. Predicted values of % student persistence to the first organic chemistry course in the series at three different time points based on logistic regression models with GChem1 course and race/ethnicity (classified as NALA: Black/African American, Hispanic/Latino, American Indian/Native American & Native Hawaiian/Pacific Islander or WA: White or Asian/Asian American) as predictors. The models controlled for term, instructor, sex of the student, and high-school GPA.

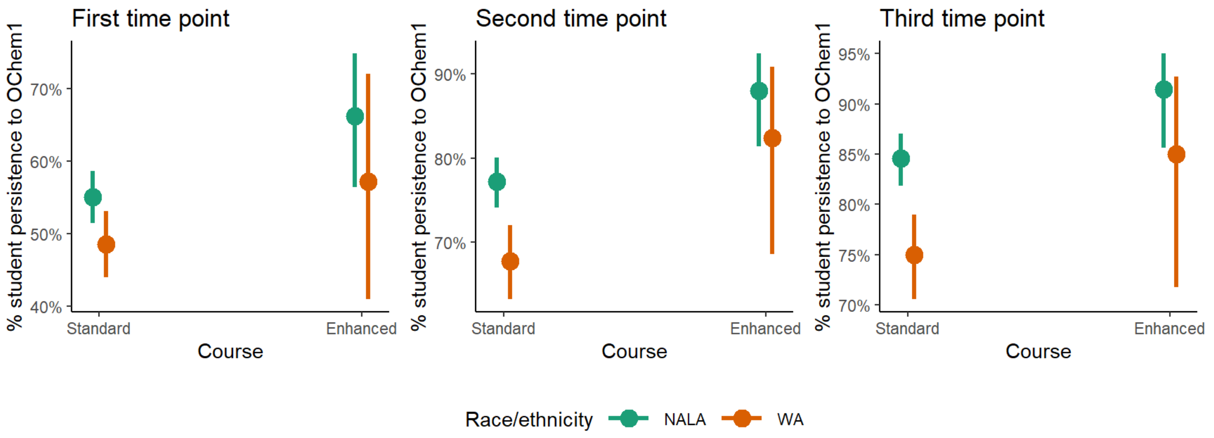

Figure S4. Predicted values of % student persistence to the first organic chemistry course in the series at three different time points based on logistic regression models with GChem1 course and sex as the predictor (classified as F: female or M: male). The models controlled for term, instructor, race/ethnicity, and high-school GPA.

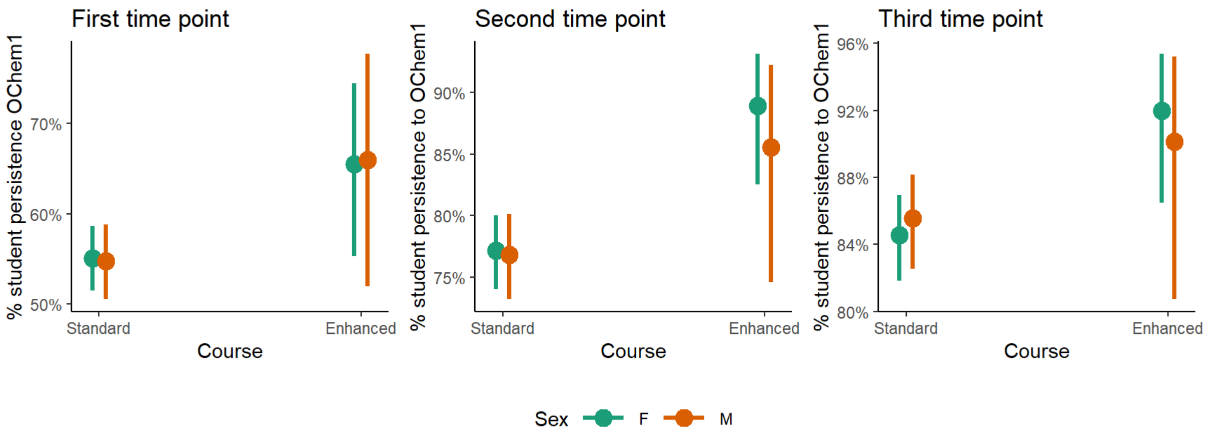

Table S6. Percent student persistence to OChem1 by race/ethnicity (classified as NALA: Black/African American, Hispanic/Latino, American Indian/Native American & Native Hawaiian/Pacific Islander or WA: White or Asian/Asian American) at different time points.

	**Standard GChem1 (Fall 2017-2020 data)**		**Standard GChem1 (Fall 2020 data)**		**Enhanced GChem1 (Fall 2020 data)**	
**% Persistence to OChem1**	NALA	WA	NALA	WA	NALA	WA
By first time point	43.4%	52.2%	49.8%	54.7%	50.0%	60.6%
By second time point	62.8%	75.9%	67.1%	76.8%	75.0%	83.4%
By third time point	71.1%	84.3%	74.6%	84.1%	77.3%	87.4%

Table S7. Percent student persistence to OChem1 by sex (classified as F: female or M: male) at different timepoints.

	**Standard GChem1 (Fall 2017-2020 data)**		**Standard GChem1 (Fall 2020 data)**		**Enhanced GChem1 (Fall 2020 data)**	
**% Persistence to OChem1**	Female	Male	Female	Male	Female	Male
By first time point	50.3%	50.6%	56.2%	47.7%	57.9%	59.0%
By second time point	73.4%	73.6%	76.2%	71.2%	83.0%	78.7%
By third time point	81.3%	82.9%	81.7%	82.8%	86.0%	83.6%

Supporting Information

Table S8. Logistic regression results for student persistence to first organic chemistry course with race/ethnicity (WA or NALA), sex (F or M), and interactions between course and race/ethnicity, and course and sex for dataset with SAT math score and high school GPA data.

	**First time point**			**Second time point**			**Third time point**		
	**log(OR)**	**95% CI**	**p-value**	**log(OR)**	**95% CI**	**p-value**	**log(OR)**	**95% CI**	**p-value**
(Intercept)	0.49	0.16, 0.82	0.003	1.6	1.2, 2.0	<0.001	2.1	1.7, 2.6	<0.001
**GChem1 Course type (reference: Standard)**									
Enhanced	0.50	-0.05, 1.1	0.078	0.88	0.20, 1.6	0.013	0.89	0.12, 1.7	0.027
**Sex (reference: Female)**									
Male	0.06	-0.10, 0.22	0.5	0.02	-0.16, 0.21	0.8	0.06	-0.16, 0.27	0.6
**Race/ethnicity (reference: WA)**									
NALA	-0.30	-0.51, -0.10	0.004	-0.31	-0.53, -0.08	0.007	-0.38	-0.63, -0.13	0.003
**Interactions with GChem1 Course type**									
Enhanced * Male	-0.20	-0.90, 0.49	0.6	-0.45	-1.3, 0.41	0.3	-0.40	-1.3, 0.57	0.4
Enhanced * NALA	0.00	-0.75, 0.75	>0.9	-0.13	-1.0, 0.77	0.8	-0.21	-1.1, 0.74	0.7
**Instructor (reference: I3)**									
I1	-0.52	-0.94, -0.11	0.013	-0.56	-1.0, -0.09	0.021	-0.61	-1.2, -0.07	0.026
I2	-0.20	-0.46, 0.05	0.12	-0.27	-0.57, 0.03	0.081	-0.15	-0.50, 0.19	0.4
**Term when GChem1 was taken (reference: 17F)**									
18F	-0.54	-0.78, -0.29	<0.001	-0.41	-0.70, -0.13	0.005	-0.30	-0.65, 0.03	0.10
19F	-0.12	-0.36, 0.13	0.4	-0.14	-0.44, 0.15	0.3	-0.26	-0.61, 0.07	0.086
20F	-0.09	-0.33, 0.16	0.5	-0.21	-0.50, 0.08	0.2	-0.31	-0.65, 0.03	0.057
**z-SAT Math score**	-0.05	-0.14, 0.04	0.3	0.16	0.06, 0.26	0.001	0.23	0.12, 0.34	<0.001
**z-High school GPA**	0.23	0.15, 0.31	<0.001	0.35	0.26, 0.43	<0.001	0.41	0.32, 0.51	<0.001
AIC	4,446			3,590			2,877		
OR = Odds Ratio, CI = Confidence Interval									

Table S9. Logistic regression results for student persistence to first organic chemistry course with race/ethnicity, sex, and interactions between course and race/ethnicity, and course and sex for larger dataset with only high school GPA data.

	First time point			Second time point			Third time point		
	**log(OR)**	**95% CI**	**p-value**	**log(OR)**	**95% CI**	**p-value**	**log(OR)**	**95% CI**	**p-value**
(Intercept)	0.36	0.07, 0.64	0.015	1.5	1.2, 1.8	<0.001	2.0	1.7, 2.4	<0.001
**GChem1 Course type (reference: Standard)**									
Enhanced	0.46	-0.01, 0.94	0.056	0.86	0.28, 1.5	0.005	0.76	0.11, 1.5	0.026
**Sex (reference: Female)**									
Male	-0.02	-0.15, 0.12	0.8	-0.02	-0.18, 0.14	0.8	0.08	-0.10, 0.27	0.4
**Race/ethnicity (reference: WA)**									
NALA	-0.26	-0.42, -0.10	0.001	-0.48	-0.65, -0.31	<0.001	-0.60	-0.79, -0.42	<0.001
**Interactions with GChem1 Course type**									
Enhanced * Male	0.03	-0.59, 0.66	>0.9	-0.29	-1.0, 0.51	0.5	-0.32	-1.2, 0.57	0.5
Enhanced * NALA	-0.12	-0.81, 0.57	0.7	0.00	-0.81, 0.85	>0.9	-0.06	-0.91, 0.84	0.9
**Instructor (reference: I3)**									
I1	-0.35	-0.71, 0.01	0.055	-0.49	-0.90, -0.08	0.02	-0.57	-1.0, -0.10	0.018
I2	-0.09	-0.32, 0.13	0.4	-0.12	-0.38, 0.13	0.4	-0.14	-0.45, 0.16	0.4
**Term when GChem1 was taken (reference: 17F)**									
18F	-0.47	-0.68, -0.26	<0.001	-0.36	-0.61, -0.12	0.004	-0.28	-0.57, 0.00	0.056
19F	-0.06	-0.27, 0.15	0.6	-0.08	-0.33, 0.17	0.5	-0.12	-0.41, 0.17	0.4
20F	-0.06	-0.28, 0.15	0.6	-0.14	-0.40, 0.11	0.3	-0.21	-0.50, 0.09	0.2
**z-High school GPA**	0.19	0.12, 0.25	<0.001	0.33	0.26, 0.40	<0.001	0.41	0.33, 0.49	<0.001
AIC	5,590			4,520			3,641		
OR = Odds Ratio, CI = Confidence Interval									

Supporting Information

Table S10. Linear regression results for student sense of belonging at the end of taking the first general chemistry course. Sense of belonging is constituted of two factors in the scale we used: *perceived belonging* and *belonging uncertainty*. Overall greater sense of belonging is indicated by higher perceived belonging scores and lower belonging uncertainty scores. Pre-belonging scores, SAT math scores and high school GPA were scaled to a mean of 0 and standard deviation of 1.

	**Perceived Belonging**			**Belonging Uncertainty**		
**Characteristic**	**Beta**	**95% CI[1]**	**p-value**	**Beta**	**95% CI[1]**	**p-value**
**Pre-measure**	0.42	0.34, 0.50	<0.001	0.28	0.18, 0.37	<0.001
**GChem1 Course type**						
Standard	—	—				
Enhanced	0.34	0.17, 0.50	<0.001	-0.32	-0.51, -0.14	<0.001
**z-SAT Math Score**	0.20	0.12, 0.28	<0.001	-0.22	-0.31, -0.13	<0.001
**z-High School GPA**	0.03	-0.06, 0.12	0.5	-0.03	-0.13, 0.07	0.6
R^2	0.300			0.171		
AIC	994			1,077		

[1] CI = Confidence Interval

Supporting Information

Table S11. Linear regression results for student sense of belonging at the end of taking the first general chemistry course for the larger dataset. Sense of belonging is constituted of two factors in the scale we used: *perceived belonging* and *belonging uncertainty*. Overall greater sense of belonging is indicated by higher perceived belonging scores and lower belonging uncertainty scores. Pre-belonging scores and high school GPA were scaled to a mean of 0 and standard deviation of 1.

	**Perceived Belonging**			**Belonging Uncertainty**		
**Characteristic**	**Beta**	**95% CI**[1]	**p-value**	**Beta**	**95% CI**[1]	**p-value**
**Pre-measure**	0.39	0.31, 0.46	<0.001	0.29	0.21,0.37	<0.001
**GChem1 Course type**						
Standard	—	—		—	—	
Enhanced	0.36	0.20, 0.51	<0.001	-0.32	-0.49, -0.15	<0.001
**z-High School GPA**	0.11	0.03, 0.19	0.007	-0.09	-0.17,0.00	0.056
R^2	0.214			0.120		
AIC	1,282			1,352		

[1] CI = Confidence Interval

Table S12. Perceived belonging and belonging uncertainty factor scores by sex and race/ethnicity at the beginning and towards the end of GChem1. Sense of belonging is constituted of two factors in the scale we used: *perceived belonging* and *belonging uncertainty*. Overall greater sense of belonging is indicated by higher perceived belonging scores and lower belonging uncertainty scores.

**Sex**	**Race/ethnicity**	**GChem1 course type**	**Pre-Perceived Belonging**	**Post-Perceived Belonging**	**Pre-Belonging Uncertainty**	**Post-Belonging Uncertainty**
			(Mean ± SD)			
Female	WA	Standard	0.04 ± 0.64	0.09 ± 0.91	0.02 ± 1.15	-0.06 ± 1.19
Female	WA	Enhanced	0.02 ± 0.71	0.32 ± 0.76	-0.08 ± 1.17	-0.33 ± 1.13
Female	NALA	Standard	-0.13 ± 0.79	-0.29 ± 1.08	0.19 ± 1.28	0.49 ± 1.37
Female	NALA	Enhanced	-0.08 ± 0.91	0.13 ± 0.56	0.36 ± 1.37	-0.01 ± 1.08
Male	WA	Standard	0.13 ± 0.59	0.16 ± 0.80	-0.23 ± 1.01	-0.01 ± 1.21
Male	WA	Enhanced	0.17 ± 0.91	0.62 ± 0.68	-0.41 ± 1.36	-0.66 ± 1.18
Male	NALA	Standard	-0.07 ± 0.86	-0.32 ± 1.05	-0.24 ± 1.11	0.31 ± 1.44
Male	NALA	Enhanced	0.04 ± 0.78	0.50 ± 0.95	0.08 ± 1.45	0.04 ± 1.45

Table S13. Linear regression results for student sense of belonging at the end of taking the first general chemistry course controlling for sex and race/ethnicity and interactions between GChem1 type and sex and race/ethnicity. Sense of belonging is constituted of two factors in the scale we used: *perceived belonging* and *belonging uncertainty*. Overall greater sense of belonging is indicated by higher perceived belonging scores and lower belonging uncertainty scores. Pre-belonging scores, SAT math scores and high school GPA were scaled to a mean of 0 and standard deviation of 1.

	**Perceived Belonging**			**Belonging Uncertainty**		
**Characteristic**	**Beta**	**95% CI**[1]	**p-value**	**Beta**	**95% CI**[1]	**p-value**
**Pre-measure**	0.42	0.34, 0.50	<0.001	0.28	0.18, 0.37	<0.001
**GChem1 Course type**						
Standard	—	—				
Enhanced	0.22	0.00, 0.45	0.049	-0.27	-0.51, -0.02	0.037
**z-SAT Math Score**	0.17	0.06, 0.27	0.001	-0.21	-0.33, -0.10	<0.001
**z-High School GPA**	0.04	-0.05, 0.13	0.4	-0.02	-0.13, 0.08	0.6
**Sex (reference: Female)**						
Male	-0.04	-0.28, 0.20	0.7	0.12	-0.15, 0.38	0.4
**Race/ethnicity (reference: WA)**						
NALA	-0.16	-0.46, 0.13	0.3	0.01	-0.33, -0.10	<0.001
**Interactions with GChem1 Course type**						
Enhanced * Male	0.31	-0.06, 0.68	0.10	-0.21	-0.62, 0.21	0.3
Enhanced * NALA	0.17	-0.24, 0.59	0.4	0.05	-0.42, 0.51	0.8
R^2	0.315			0.171		

[1] CI = Confidence Interval

Table S14. Linear regression results for student sense of belonging at the end of taking the first general chemistry course controlling for sex and race/ethnicity and interactions between GChem1 type and sex and race/ethnicity. Sense of belonging is constituted of two factors in the scale we used: *perceived belonging* and *belonging uncertainty*. Overall greater sense of belonging is indicated by higher perceived belonging scores and lower belonging uncertainty scores. Pre-belonging scores and high school GPA were scaled to a mean of 0 and standard deviation of 1.

	**Perceived Belonging**			**Belonging Uncertainty**		
**Characteristic**	**Beta**	**95% CI[1]**	**p-value**	**Beta**	**95% CI[1]**	**p-value**
**Pre-measure**	0.38	0.30, 0.45	<0.001	0.29	0.20, 0.38	<0.001
**GChem1 Course type**						
Standard	—	—				
Enhanced	0.26	0.05, 0.46	0.014	-0.23	-0.46, -0.01	0.037
**z-High School GPA**	0.10	0.01, 0.18	0.022	-0.06	-0.15, 0.03	0.2
**Sex (reference: Female)**						
Male	-0.01	-0.24, 0.22	>0.9	0.06	-0.19, 0.31	0.6
**Race/ethnicity (reference: WA)**						
NALA	-0.32	-0.58, -0.05	0.019	0.29	0.00, 0.57	0.048
**Interactions with GChem1 Course type**						
Enhanced * Male	0.25	-0.10, 0.61	0.2	-0.19	-0.57, 0.20	0.3
Enhanced * NALA	0.17	-0.23, 0.58	0.4	-0.06	-0.49, 0.38	0.8
R^2	0.236			0.137		

[1] CI = Confidence Interval

Figure S5. Grade distribution in Standard and Enhanced GChem1 for the dataset from Fall 2017, Fall 2018, Fall 2019, & Fall 2020. Note that Enhanced GChem1 was only taught in Fall 2020.

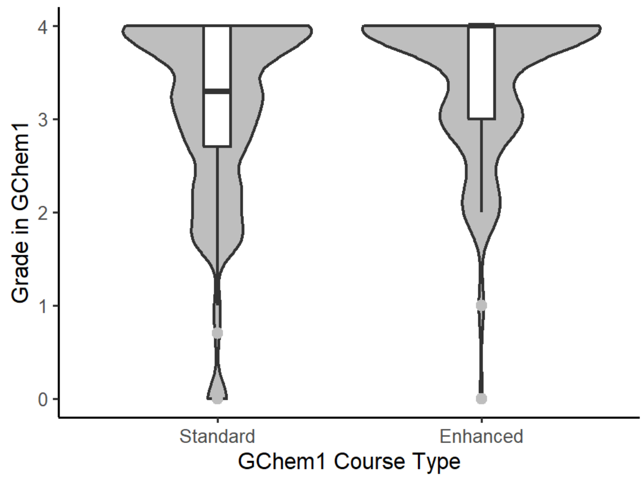

Figure S6 Correlation plots for A) Pre-survey data and B) Post-survey data for the sense of belonging scale. Items sob1-sob4 form the "perceived belonging" factor and items sob5-sob6 form the "belonging uncertainty" factor and the two are inversely related to each other.

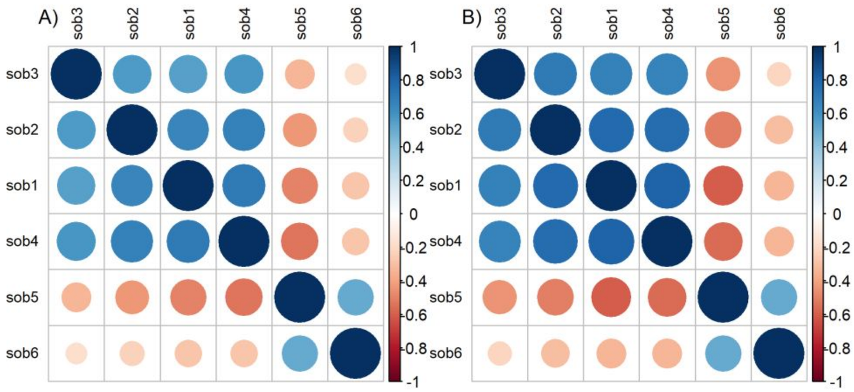

Following the work of others using the same belonging survey, we hypothesized that the survey data would have a two-factor structure with items 1-4 factoring together as "Perceived Belonging" and items 5-6 factoring together as "Belonging Uncertainty".[1,2] To check this hypothesis for our data, we first calculated and plotted correlation matrices for pre- and post- responses to the six items (Figure S6 above). These plots were consistent with the hypothesized two-factor structure. Because previous studies have demonstrated the two-factor structure of this scale, we went ahead and ran a confirmatory factor analysis. First, we checked the assumptions for confirmatory factor analysis by calculating factorability using Kaiser's measure of sampling adequacy and examining skewness and kurtosis. We found factorability values of 0.84 for the pre-survey data and 0.87 for the post-survey data, which are above the 0.6 cut off suggested for good factorability.[3] Skewness and kurtosis values were lower than 2 for both pre- and post-survey data, except item 3 in the post-survey data. Using Mardia test of multivariate skew and kurtosis, we found some evidence for multivariate non-normality. Therefore, for our CFA model, we used maximum likelihood estimation with robust standard errors to handle non-linearity in our data.

Table S15. Results from the mediation analyses using 1000 quasi-Bayesian Monte Carlo simulations to calculate confidence intervals and statistical significance. Mediation effect indicates the amount of the association of GChem1 course type with persistence to OChem1 that is mediated by students' grade received in GChem1 course. Direct effect indicates the amount of the association of GChem1 course type with persistence not related to GChem1 grades. Proportion mediated shows the proportion of total association of GChem1 course type with persistence that can be attributed to GChem1 grades.

	**First timepoint**			**Second timepoint**			**Third timepoint**		
	Estimate	95% CI	p-value	Estimate	95% CI	p-value	Estimate	95% CI	p-value
Average Causal Mediation Effect	0.06	0.04-0.08	<0.001	0.06	0.04-0.08	<0.001	0.05	0.03-0.06	<0.001
Average Direct Effect	0.05	-0.06-0.15	0.39	0.03	-0.06-0.11	0.51	0.008	-0.07-0.07	0.77
Proportion mediated	0.52	-0.89, 4.39	0.058	0.60	-1.46, 3.19	0.056	0.71	-6.41, 5.40	0.15

References

(1) Fink, A.; Frey, R. F.; Solomon, E. D. Belonging in General Chemistry Predicts First-Year Undergraduates' Performance and Attrition. *Chem. Educ. Res. Pract.* **2020**, *21* (4), 1042–1062. https://doi.org/10.1039/D0RP00053A.

(2) Edwards, J. D.; Barthelemy, R. S.; Frey, R. F. Relationship between Course-Level Social Belonging (Sense of Belonging and Belonging Uncertainty) and Academic Performance in General Chemistry 1. *J. Chem. Educ.* **2022**, *99* (1), 71–82. https://doi.org/10.1021/acs.jchemed.1c00405.

(3) Knekta, E.; Runyon, C.; Eddy, S. One Size Doesn't Fit All: Using Factor Analysis to Gather Validity Evidence When Using Surveys in Your Research. *Factor Anal.* **2019**, 17. https://doi.org/10.1187/cbe.18-04-0064.
